# A highly efficient scheme for library preparation from single-stranded DNA

**DOI:** 10.1038/s41598-023-40890-3

**Published:** 2023-08-25

**Authors:** Fumihito Miura, Hideaki Kanzawa-Kiriyama, Osamu Hisano, Miki Miura, Yukiko Shibata, Noboru Adachi, Tsuneo Kakuda, Ken-ichi Shinoda, Takashi Ito

**Affiliations:** 1 Department of Biochemistry, Kyushu University Graduate School of Medical Sciences, 3-1-1 Maidashi, Higashi-Ku, Fukuoka, 812-8582 Japan; 2 Department of Anthropology, National Museum of Nature and Science, 4-1-1 Amakubo, Tsukuba, Ibaraki 305-0005 Japan; 3 Department of Clinical Radiology, Kyushu University Graduate School of Medical Sciences, 3-1-1 Maidashi, Higashi-Ku, Fukuoka, 812-8582 Japan; 4 Department of Legal Medicine, Interdisciplinary Graduate School of Medicine and Engineering, University of Yamanashi, 1110 Shimokato, Chuo, Yamanashi 409-3898 Japan; 5 National Museum of Nature and Science, 4-1-1 Amakubo, Tsukuba, Ibaraki 305-0005 Japan

**Keywords:** Diagnostic markers, DNA, Catalytic DNA, Catalytic RNA, Ligases, Bioinformatics, Genomic analysis, Next-generation sequencing

## Abstract

Although methods for sequencing library preparation from double-stranded DNA are well established, those from single-stranded DNA (ssDNA) have not been well studied. Further, the existing methods have limitations in efficiency and yield. Therefore, we developed a highly efficient procedure for sequencing library preparation from ssDNA. In this method, the first adaptor tagging of ssDNA is performed using terminal deoxyribonucleotidyl transferase (TdT)-assisted adenylate connector-mediated ssDNA (TACS) ligation, which we reported recently. After complementary strand synthesis using the adaptor-tagged ssDNA, second adaptor tagging via Vaccinia virus topoisomerase I (VTopoI or TOPO)-based adaptor ligation is performed. With additional steps for degradation, repression, and removal of the adaptor dimer, the proposed TACS-TOPO scheme realizes adaptor dimer-free sequencing library preparation from ssDNA samples of 24 pg. The TACS-TOPO scheme was successfully applied to cell-free DNA analysis with amplification-free library preparation from 50 µL of human serum. A modified TACS-TOPO scheme was also applied to DNA extracted from ancient human bones, bringing two to eight times more library yields than those using a conventional library preparation protocol. The procedures for preparing VTopoI and its complex with a double-stranded oligonucleotide adaptor are also described. Overall, the proposed TACS-TOPO scheme can facilitate practical and sensitive sequencing analysis of ssDNA.

## Introduction

Although many efficient methods are available for sequencing library preparation from double-stranded DNA (dsDNA)^1–3^, limited methods are reported for single-stranded DNA (ssDNA). Only three major principles for sequencing library preparation from ssDNA have been established to date. The first approach involves terminal deoxyribonucleotidyl transferase (TdT)-mediated homopolymeric tailing to the 3′-end of ssDNA; the tail is then used as the priming site to convert the target ssDNA to dsDNA, which is followed by the second adaptor tagging with T4 DNA ligase^4^. TdT-mediated library preparation is highly efficient, and some manufacturers provide library preparation kits based on this principle. However, a lengthy tail can constitute an obstacle for downstream sequencing analysis.

The second method is based on ssDNA ligation catalyzed by RNA ligase, which joins a 5′-phosphorylated adaptor to the 3′-end of ssDNA. For example, Meyer's group established a method based on CircLigase II^5,6^. After adaptor ligation, ssDNA is converted to dsDNA, followed by second adaptor tagging by T4 DNA ligase. Although RNA ligase can connect two ssDNA molecules, the reaction efficiency is limited to less than a few percent. Therefore, the yield of library preparation using RNA ligase is rather limited.

The third approach uses T4 DNA ligase for ssDNA ligation^7^. A double-stranded adaptor with a single-stranded random hexamer at one end is used in this method. If the random hexamer is compatibly hybridized to the end of the target ssDNA, the nick at the partially double-stranded end of the adaptor can be sealed with T4 DNA ligase. This splinted ligation-based procedure is called ssDNA2.0 and shows efficiency superior to the CircLigase II-based method^7^. Although both RNA ligase- and T4 DNA ligase-based methods can produce clean connections with the target ssDNA, their low efficiencies in library preparation remain to be addressed.

To improve sequence library preparation from ssDNA, we developed a technique for the efficient adaptor tagging of ssDNA, termed terminal deoxyribonucleotidyl transferase (TdT)-assisted adenylate connector-mediated single-stranded (ss) DNA (TACS) ligation^8^. In TACS ligation, target ssDNA is first tailed with a few adenylates using TdT in a process called ribotailing, followed by RNA ligase-mediated adaptor tagging of the ribotailed ssDNA. Ribotailing of the 3′-end of ssDNA allows it to behave as RNA, which greatly enhances the ssDNA ligation efficiency of RNA ligases. Accordingly, TACS ligation allows adaptor-tagging of more than 80% of the 3′-end of target ssDNA^8^. Notably, the tailing reaction of TdT with ribonucleotide is autoinhibited after extending 1–3 nucleotides^9^, which results in only minimal interruption of the downstream sequencing analysis.

TACS ligation has recently been applied to the analysis of short ssDNA in the cell-free fraction of human blood^10^. After the adaptor tagging of cell-free DNA (cfDNA) via TACS ligation, the target ssDNA was converted to dsDNA, followed by additional adaptor tagging using T4 DNA ligase (TACS-T4 scheme). The yield of the TACS-T4 scheme is several times higher than that of CircLibase II and T4 DNA ligase-based methods^10^.

Although the TACS-T4 scheme showed improved library yields, the protocol has some practical problems, including inefficient second adaptor tagging and non-negligible adaptor dimer formation^10^. Although the second ligation efficiency could be enhanced through reaction optimization, this could simultaneously increase adaptor dimer formation. Adaptor dimer formation could be avoided if the unreacted adaptor is removed before the second ligation step. However, an extra purification step would inevitably result in losing precious DNA. Further, complete removal of the unreacted adaptor is very difficult, especially when the size difference between the adaptor and product is small. Therefore, we aimed to establish an alternative method with reduced dimer formation and enhanced second adaptor tagging.

To this end, we considered whether Vaccinia virus topoisomerase I (VTopoI)-based adaptor tagging would work better than T4 DNA ligase because VTopoI-based ligation has a directionality differing from that of ligation with T4 DNA ligase: most DNA/RNA ligases connect the 5′-phosphorylated end to the 3′-hydroxyl terminal of target DNA, whereas VTopoI activates the 3′-phosphorylated end to connect the 5′-hydroxyl end of target DNA. Owing to this difference in substrate specificity, VTopoI could never connect its substrate DNA to the 5′-phosphorylated, unreacted adaptor used in the first adaptor tagging step with TACS ligation.

The gene encoding VTopoI was first identified by Stewart Shuman in 1987^11^. Since then, he and his colleagues have extensively studied its enzyme properties^12–20^. VTopoI was reported as a tool for molecular cloning in 1994^21^; later, this technology was commercialized by Invitrogen (currently Thermo Fisher Scientific). VTopoI has been used as an efficient enzyme to clone DNA fragments into vectors in the so-called TOPO cloning technology. Once activated by VTopoI, the vector can connect to the target DNA, and ligation occurs rapidly, efficiently, and specifically. Based on these properties, TOPO cloning has been considered a good choice for initiating molecular cloning. Although the characteristics of VTopoI in ligation seem attractive for developing new technologies, limited studies have examined the use of VTopoI as an enzyme for ligation^22^.

In the present study, we investigated the use of VTopoI to prepare libraries for next-generation sequencing from short ssDNA combined with TACS ligation. We describe methods for the purification of VTopoI, formation of the VTopoI-oligonucleotides (ODN) complex (VOC), purification of the VOC, and its application for sequencing library preparation from ssDNA (TACS-TOPO scheme).

## Materials and methods

### Vector construction

The gene encoding VTopoI (GenBank: L13447.1) was synthesized by Eurofins Genomics Inc (Tokyo, Japan), with codon optimization for *Escherichia coli* K-12, exclusion of internal BamHI and EcoRI sites, and their inclusion at the 5′- and 3′-ends of the DNA, respectively (Supplementary Sequences). The BamHI-EcoRI fragment of the gene was subcloned into the BamHI-EcoRI site of pColdTF, pColdI, and pET28a. To produce T4 polynucleotide kinase (T4 PNK), we used polymerase chain reaction (PCR) to amplify the pseT gene of T4 phage from genomic DNA (T4 GT7 DNA, Nippon Gene, Tokyo, Japan) with BamHI and EcoRI sites at the 5′ and 3′ ends, respectively (Supplementary Sequences), and cloned it into the BamHI-EcoRI site of pColdI. These constructs were introduced into T7Express (New England Biolabs, Ipswich, MA) and cultivated in Luria–Bertani broth (LB) medium (1% (w/v) tryptone, 0.5% (w/v) yeast extract, and 0.5% (w/v) sodium chloride) with 100 µg/mL carbenicillin (for pColdTF and pColdI vector, Nacalai Tesque, Kyoto, Japan) or 50 µg/mL kanamycin (for pET28a, Fujifilm Wako Chemicals, Tokyo, Japan).

### Enzyme purification

*Cultivation of bacterial cells.* Bacterial cells transformed with one of the expression vectors were inoculated into 3 mL of LB medium with antibiotics and cultivated overnight at 37 °C with vigorous shaking. The seed culture was transferred into a 2-L Erlenmeyer flask with baffles, containing 1 L of LB medium with antibiotics and further cultivated at 37 °C for 4 h with shaking. For pColdI and pColdTF, the flask was cooled in ice-cold water for 30 min, and protein expression was induced by adding 238 mg of Isopropyl-β-D-thiogalactopyranoside (IPTG). For pET28a, protein expression was induced by adding 238 mg of IPTG. The flasks were incubated at 16 °C overnight (pColdI and pColdTF) or 37 °C for 4 h (pET28a) with vigorous shaking. The cells were collected by centrifugation at 2500×*g* for 15 min and stored at − 80 °C until use.

*Column purification of recombinant proteins.* Bacterial cells were dissolved in 20 mL of HisTrap buffer A (20 mM sodium phosphate, pH 7.5, 800 mM NaCl, 20 mM imidazole, 1 mM dithiothreitol (DTT), and 10% glycerol) containing protease inhibitor cocktail (Nacalai Tesque) and disrupted by sonication. The lysate was cleared by centrifugation at 15,000×*g* for 15 min and filtration through a 0.45 µm syringe filter. First-round chromatographic purification was performed using a 5 mL HisTrap HP column (Cytiva, Marlborough, MA) with HisTrap buffer A and HisTrap buffer B (20 mM sodium phosphate, pH 7.5, 800 mM NaCl, 200 mM imidazole, 1 mM DTT, and 10% glycerol). The fraction containing the target protein was then diluted tenfold with Heparin buffer A (50 mM sodium phosphate, pH 7.5, 100 mM NaCl) and subjected to further purification using a 5 mL HiTrap Heparin HP column (Cytiva) with Heparin buffer A and Heparin buffer B (50 mM sodium phosphate, pH 7.5, 1 M NaCl). The target protein fraction was then collected and buffer-exchanged with stock buffer (50 mM Tris–HCl, pH 8.0, 300 mM NaCl) using the HiPrep 26/10 Desalting column. These chromatographic purifications were performed on the AKTA start system (Cytiva). We used the default template programs of affinity, ion exchange, and desalting for the HisTrap, HiTrap Heparin, and HiPrep Desalting processes, respectively.

*Finalization and storage of the purified proteins.* Finally, the protein was concentrated by ultrafiltration using an Amicon Ultra-4 30 K device (Millipore). The concentration of VTopoI was adjusted to 200 µM and stored at 4 °C until use. The molar concentration of VTopoI was determined by measuring the optical absorbance at 280 nm and calculating the molar absorption coefficient using the CLC main workbench (Qiagen, Hilden, Germany). For T4 PNK, the protein concentration was adjusted to 2 mg/mL (Bradford method standardized with bovine serum albumin), and the solution was then supplemented with equivolume glycerol and stored at − 20 °C. The activities of the purified VTopoI proteins were assayed as described in the Supplementary Methods.

### Formation of the VTopoI-ODN complex and its purification

The ODNs used in this study are summarized in Supplementary Table S1. A 1 mL reaction containing 10 mM Tris–HCl, pH 8.0, 160 mM sodium chloride, 10 µM TOPO-adaptor-F, 10 µM TOPO-adaptor-R (either of TA or blunt), and 10 µM TOPO-adaptor-Ext was prepared in a 1.5-mL test tube. The reaction mixture was incubated at 95 °C for 3 min, followed by 55 °C for 5 min, and kept at 37 °C. Then, 1 mL of the oligo mixture, 1 mL of 5 × Topo activation buffer (100 mM Tris–acetate, pH 8.0, 250 mM potassium acetate, and 50 mM magnesium acetate), 100 µL of 100 mM adenosine triphosphate (ATP), and 100 µL of 1 mg/mL T4 PNK were added to a 15-mL centrifuge tube, and the total volume was adjusted to 5 mL with water. After incubation at 37 °C for 60 min, the reaction mixture was cooled to 30 °C, and 250 µL of 200 µM VTopoI was added. The reaction was incubated at 30 °C for 30 min.

The reaction mixture was then loaded on a 1-mL HiTrap Heparin HP column and separated using Heparin buffers A and B as described above. The fractions were analyzed using both sodium dodecyl sulfate (SDS)-polyacrylamide gel electrophoresis (PAGE) and agarose gel electrophoresis. For SDS-PAGE, samples were run on a Mini-PROTEAN Any kD gel (BioRad Laboratories), and the gel was stained with Bullet CBB Stain One (Nacalai Tesque). For agarose gel electrophoresis, 10 µL of each fraction was taken, supplemented with 1 µL of 10% SDS solution, and 1 µL of the mixture was loaded into the E-Gel Ex (2%) gel. Two major peaks appeared on the heparin column, and the fraction containing the VTopoI-ODN complex eluted earlier than the unreacted VTopoI. The VTopoI-ODN complex-containing fraction was collected and adjusted to a DNA concentration of 150 ng/µL as measured using the Qubit dsDNA HS kit. If necessary, the solution was concentrated with an Amicon Ultra-4 30 K device and stored at 4 °C until use.

### Isolation of cfDNA from human serum

All human sera and plasmas used in the current study were purchased from commercial sources and are summarized in Supplementary Table S2. The procedure described below represents cfDNA preparation from 100 µL of human serum. However, the serum or plasma volume used differed depending on the experimental purpose (the starting serum/plasma volume is indicated in each data). In such cases, the mixing ratios of the reagents were maintained.

To a 1.5-mL microcentrifuge tube (Eppendorf), 100 µL of human serum, 400 µL of 10 mM Tris–HCl, pH 8.0, 5 µL of 500 mM ethylenediaminetetraacetic acid (EDTA), 1.5 µL of 5 M NaCl, 10 µL of 10% (w/v) SDS, and 10 µL of 20 mg/mL proteinase K (Qiagen) were added and incubated at 50 °C for 1 h. After the protease digestion, extractions were performed twice with TE Saturated Phenol (Nippon Gene) and once with phenol–chloroform-isoamyl alcohol (25:24:1) (Nacalai Tesque). Then, the aqueous phase was transferred to a new tube, supplemented with 50 µL of 3 M sodium acetate, pH 5.4, and 1100 µL of isopropanol. The mixture was centrifuged at 15,000 × *g* for 5 min, and the supernatant was discarded by decantation. Then, the DNA pellet was rinsed with 70% (v/v) ethanol and dissolved in 10 mM Tris–HCl, pH 8.0.

### TACS-T4 scheme

Library preparation from cfDNA using the TACS-T4 scheme was performed as described previously^10^ with some modifications: we repeated the solid-phase reversible immobilization (SPRI)-based adaptor-dimer removal only twice, whereas this removal was repeated five times in the previous study.

### TACS-TOPO scheme

*TACS ligation.* A solution containing the target ssDNA was mixed with 2.5 µL of 10 × TACS buffer [500 mM 2-(4-(2-Hydroxyethyl)piperazin-1-yl)ethanesulfonic acid (HEPES)-KOH, pH 7.5, 50 mM MgCl_2_, 5% (v/v) Triton X-100] and 1 µL of Shrimp Alkaline Phosphatase (rSAP) (New England Biolabs, Ipswich, MA); the total volume was then adjusted to 11 µL with ddH2O. The reaction mixture was incubated at 37 °C for 15 min and 95 °C for 5 min. Next, 10 µL of 50% (w/v) polyethylene glycol (PEG) 6000, 1 µL each of 10 mM ATP, 25 µM adaptor for ver. X (X representing the protocol version number 1–5, see Supplementary Table S1), 1 µL of 15 U/μL TdT (Takara Bio Inc, Shiga Japan), and 1 µL of 2 mg/mL TS2126 RNA ligase were added. TS2126 RNA ligase was prepared as described previously^10^. Note that TS2126 RNA ligase can be substituted with CircLigase II ssDNA ligase from Lucigen (Middleton, WI). The reaction was incubated at 37 °C for 15 min, 65 °C for 30 min, and 95 °C for 5 min.

*Replication of adaptor-tagged ssDNA.* The adaptor-tagged ssDNA solution was mixed with 14 µL of ddH2O, 5 µL of 10 × ExTaq buffer (Takara Bio), 4 µL of 2.5 mM dNTPs, 1 µL of 50 µM replicating primer (Supplementary Table S1), and 1 µL of 5 unit/µL hot start GeneTaq (Nippon Gene) followed by incubation at 95 °C for 3 min, 45 °C for 5 min, 55 °C for 5 min, and 65 °C for 5 min.

*VOC-mediated second adaptor tagging.* After complementary strand synthesis, 5 µL of 500 mM EDTA (Nacalai Tesque, Kyoto, Japan) and 1 µL of 150 mg/mL VOC (TA type) were added to the reaction mixture and incubated at 25 °C for 15 min.

*Purification of the library.* After the second adaptor tagging, the reaction mixture was supplemented with 35 µL of Buffer B2 (3 M guanidine hydrochloride, 20% Tween 20) and 5 µL of Proteinase K (Qiagen) and incubated at 50 °C for 15 min. Then, 2 µL of Sera-Mag Carboxylate Magnetic Beads (Cytiva, Marlborough, MA) and 100 µL of isopropanol were mixed. After incubation at room temperature for 5 min, the magnetic particles were collected on a magnetic stand, and the supernatant was removed. The beads were washed twice with 200 µL of a solution containing 32% (v/v) PEG400, 1 M NaCl, and 50 mM Tris–HCl, pH 8.0. Finally, the beads were rinsed with 70% (v/v) ethanol, and the purified DNA was eluted with 25 µL of 10 mM Tris–Acetate, pH 8.0.

*Uracil DNA glycosylase (UDG) and apurinic/apyrimidinic Endonuclease 1 (APE 1) treatment, nick filling, and PCR amplification.* To the purified library, 5 µL of 10 × ExTaq Buffer, 4 µL of 2.5 mM dNTPs, 1 µL of 20 µM Amplification Univ (Supplementary Table S1), 1 µL of 20 µM Amplification Index-X (X referring to one of the indexes, see Supplementary Table S1), 1 µL of UDG (New England Biolabs), and 1 µL of APE 1 (New England Biolabs) were added. UDG and APE 1 were used only with the TACS-TOPO ver. 4 protocol (see “Results” section). After adjusting the reaction volume to 50 µL with water, the reaction was incubated at 37 °C for 15 min (if UDG and APE 1 were included), 72 °C for 5 min, and 95 °C for 1 min, followed by cycles of 3-step incubations at 95 °C for 15 s, 55 °C for 30 s, and 72 °C for 1 min. The number of cycles used is indicated in the presented data. When necessary, the reaction mixture was purified with 90 µL of AxyPrep MAG PCR clean (Corning, Corning, NY) according to the manufacturer's instructions. The purified DNA was eluted in 21 µL of 10 mM Tris–Acetate, pH 8.0.

### Ancient human bone DNA

The ancient human DNA used in the current study was extracted as described previously^23^. Bones from the Inome cave site^24^ and Shimekake site^25^ were used. The ancient DNAs used in the current study are summarized in Supplementary Table S3. Because the concentrations of the purified ancient DNA were weak and difficult to estimate, we performed sequencing library preparations without measuring the concentrations.

### Library preparation with commercial kits

Sequencing library preparations based on the conventional methods targeted to dsDNA were performed using either the ThruPlex DNA Seq kit (Takara Bio Inc.) or KAPA Hyper Prep Kit (Kapa Biosystems, Cape Town, South Africa).

*ThruPlex DNA Seq kit*. Starting from an indicated amount of DNA, end polishing and adaptor ligation were performed following the manufacturer's instructions. Then, a 50-µL PCR mixture was prepared following the manual provided by the manufacturer. The reaction was incubated at 72 °C for 3 min, 84 °C for 2 min, and 98 °C for 2 min, followed by four cycles of three-step incubations at 98 °C for 20 s, 67 °C for 20 s, and 72 °C for 40 s, and eight cycles of two-step incubation at 98 °C for 20 s and 72 °C for 50 s. The reaction mixture was supplemented with 50 µL of AxyPrep MAG PCR and incubated at room temperature for 5 min. The magnetic beads were then rinsed with 70% (v/v) ethanol, and purified DNA was eluted in 25 µL of 10 mM Tris–Acetate, pH 8.0. The purified library was stored at − 20 °C until use.

*Kapa Hyper Prep Kit.* A 50-µL solution containing sample DNA was supplemented with 7 µL of End repair & A-tailing buffer and 3 µL of End repair & A-tailing enzyme, incubated sequentially at 20 °C for 30 min and 65 °C for 30 min. The reaction was supplemented with 5 µL of 300 nM Kapa universal adaptor, 30 µL of ligation buffer, 5 µL of water, and 10 µL of DNA ligase and incubated at 20 °C for 15 min. Then, the reaction was supplemented with 88 µL of AMPure XP beads (Beckman) and incubated at room temperature for 10 min. The beads were collected on a magnetic stand to remove the supernatant, rinsed twice with 80% (v/v) ethanol, and the purified DNA was eluted with 20 µL of 10 mM Tris–Acetate, pH 8.0. A PCR reaction was set up by mixing the 20 µL of purified DNA, 25 µL KAPA HiFi HotStart Uracil + ReadyMix (2X), and 5 µL of primer mix from KAPA UDI primer mixes, and thermal cycling was performed with the following conditions: a pre-PCR incubation at 98 °C for 45 s; indicated cycles of three-step incubations at 98 °C for 15 s, 60 °C for 30 s, and 72 °C for 30 s; and a post-PCR incubation at 72 °C for 1 min.

### Library quantitation and sequencing

The molar concentration of the sequencing library was determined using the library quantification kit from Takara Bio Inc. Small-scale sequencing was performed with MiSeq using the MiSeq Reagent Kit v2 nano kit (300 cycles) in the paired-end mode with 2 × 151 cycles. For large-scale sequencing, the paired-end mode with 2 × 151 cycles using the HiSeq X Ten was performed by Macrogen Japan Corp. (Tokyo, Japan).

### Sequence analysis

*cfDNA analysis.* cfDNA reads were analyzed as described previously^10^. Nucleosome-protected DNA (NPD) was defined as DNA fragments of 147–190-nt length. In contrast, cell-free 3S (short single-stranded) DNA (C3D) was defined as fragments of 35–75 nt.

*Ancient samples.* Reads were first preprocessed using fastp^26^ to trim and merge paired-end reads into a single sequence. The merged reads were then mapped on the human reference genome using the Burrows-Wheeler Alignment tool (BWA) with the ‘mem’ option^27^. Finally, the exported Sequence Alignment/Map (SAM) files were analyzed using mapDamage^28,29^. The details of the analysis parameters are provided in the Supplementary Methods.

## Results

### Purification of active VTopoI

We artificially synthesized VTopoI and subcloned it into three expression vectors. We used pColdTF, pColdI, and pET28a for the bacterial expression of VTopoI and successfully induced the protein expression (Supplementary Figure S1A). Nickel resin purifications were then performed using the HisTrap HP column (Supplementary Figure S1A). The elute from the nickel column was further fractionated using heparin resin (Supplementary Figure S1B,C). After purification, the protein purities were estimated to be more than 95% for all the vector systems used (Supplementary Figure S1D). The activities of the purified protein were assayed for relaxing the supercoiled plasmid^11^. All the purified recombinant proteins showed activity (Supplementary Figure S1E). Thus, we successfully purified the active VTopoI proteins. Since the protein yield obtained using the pET28a system was low, we used VTopoI produced with pColdI and pColdTF for further development.

### Formation and purification of the VTopoI-ODN complex and its use

**Figure 1:**
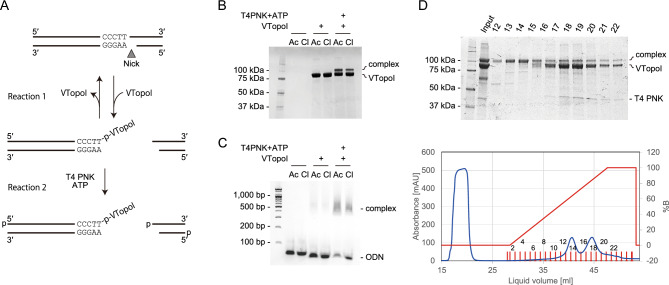
Formation and purification of the VTopoI-oligonucleotide complex (VOC). (**A**) Scheme of complex formation. The formation of VOC is an equilibrium reaction. Phosphorylation of released DNA effectively increases the amount of complex formed. (**B**,**C**) An example of VOC formation. The effects of T4 PNK and buffer compositions on complex formation are shown. Gel images of SDS-PAGE (**B**) and agarose gel electrophoresis with E-Gel Ex (**C**) are shown. VTopoI expressed with pColdTF was used for VOC formation. The expected VTopoI protein expressed with the pColdTF vector is 91.0 kDa. (**D**) Purification of the VOC with Heparin column chromatography. SDS-PAGE (top) and chromatogram (bottom) analyses of the fractions are shown. The blue and red lines in the chromatogram indicate absorbance at 280 nm and the solution B content, respectively. The numbers on the gel image and the chromatogram indicate the fraction numbers. The VOC is eluted earlier than VTopoI. The original gel images for B, C, and D are provided in Supplementary Figure S5.

VTopoI specifically recognizes the pentanucleotide sequence 5′-(C/T)CCTT-3′ on the substrate dsDNA and cleaves the 3′-end of the sequence to form a covalent bond with the 3′-phosphate group^13,21^. This single-strand cleavage leads to the release of supercoiling. After substrate DNA relaxation, the reverse reaction occurs to rejoin the cleaved strand. In the case that the substrate DNA has a nick or gap at the opposite position of the cleavage site, the downstream DNA of the recognition sequence is released from the intermediate complex to form an activated VTopoI-DNA complex (Fig. 1A, reaction 1). If a DNA molecule with a compatible end is available, VTopoI executes the reverse reaction to ligate with the DNA molecule^21^. This reaction is quick and efficient and forms the basis of Topo-based ligation.

As the reaction between the 5′-(C/T)CCTT-3′-containing substrate DNA and VTopoI is bidirectional, the ratio of the VTopoI-DNA complex to the free VTopoI is defined by the concentrations of these molecules (Fig. 1A, reaction 1). Thus, prevention of the reverse reaction would effectively increase the formation of the covalent intermediate of VTopoI and DNA (Fig. 1A, reaction 2). To achieve this, phosphorylation of the released DNA is useful because the released DNA cannot be rejoined after 5′-phosphorylation (Fig. 1A, reaction 2). As expected, the formation of the covalent intermediate of VTopoI with ODN (VOC) was enhanced when the reaction was supplemented with T4 PNK and ATP (Fig. 1A–C). The components of the solution also affect the amount of the VOC formed. For unknown reasons, the acetate ion was more effective for complex formation than the chloride ion (Fig. 1B,C). We also found that VTopoI produced from the pColdI construct tends to precipitate during VOC formation (not shown); therefore, we used the pColdTF-based VTopoI for further development. Based on these and other investigations, we selected one of the efficient conditions for VOC formation (see “Materials and methods” section).

After VOC formation, the solution still contains unreacted ODN, VTopoI, and T4 PNK, which should be removed to prevent side reactions in the downstream operations. We found that heparin affinity chromatography was effective for this purpose as well (Fig. 1D). Unreacted ODNs are negatively charged and cannot bind to the negatively charged heparin resin. The VOC can bind to the heparin column via VTopoI, but it is eluted at lower salt concentrations than the unreacted VTopoI and T4 PNK (Fig. 1D). Therefore, the separation of VOC from other unreacted materials can be achieved via a single chromatographic operation with a heparin column. After separation, the VOC can easily be concentrated by ultrafiltration. The purified VOC showed reasonably high ligation activity with a model dsDNA (see below); this activity could be maintained for over a few months with storage at 4 °C.

### VOC realizes efficient adaptor tagging of 5′-unphosphorylated dsDNA ends

**Figure 2:**
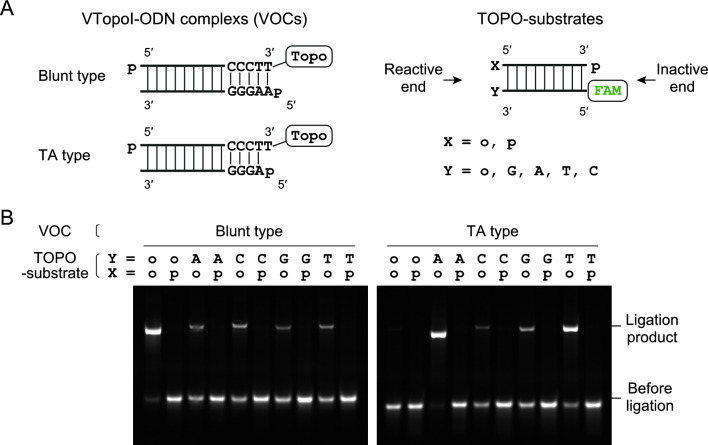
Substrate specificities of the VTopoI-oligonucleotide complex (VOC). (**A**) Structures of VOCs (left) and TOPO substrates (right). (**B**) Images of gel electrophoresis after mixing the VOC and TOPO substrates. Blunt (right) and TA (left) types of VOC were investigated for their reactivity with substrates with or without the 5′-phosphate and one base protrusions. If ligation occurs, the band shifts upward. The symbols indicate the following: o, hydroxyl end; p, phosphorylated end; A, C, G, or T, terminal nucleotide attached at the 5′-end of the substrate. For experimental details, see Supplementary Methods. The original gel images for B are provided in Supplementary Figure S6.

We next investigated the specificity and efficiency of adaptor tagging by VOC-mediated ligation. We prepared double-stranded ODNs (TOPO-substrates) as model substrates for this experiment. The TOPO-substrates were designed to accept VOC-mediated ligation at one end (reactive end), whereas the other end was blocked with 5′-FAM and 3′-phosphorylation (inactive end) (Fig. 2A). Blunt and 3′-protruded ends with A, C, G, or T were prepared at the reactive end to see the stringency of ligation (Fig. 2A). Further, to investigate the specificity of VOC to the phosphorylation states of the 5′-end, we prepared both 5′-hydroxyl and 5′-phosphorylated ends for the reactive end (Fig. 2A). Blunt end and TA ligation types were prepared for the VOC (Fig. 2A).

We investigated the ligation efficiencies of all combinations of TOPO-substrates and VOCs (Fig. 2B). Both VOCs, i.e., the blunt and TA types, showed strong ligation activity with the TOPO-substrate of the compatible ends. The reaction was nearly complete within 15 min when sufficient VOCs were supplied (Fig. 2B and Supplementary Methods). Although the VOCs showed strong activity at the 5′-hydroxyl end, they never connected the adaptor to the 5′-phosphorylated end (Fig. 2B). Adaptor ligation to the TOPO-substrate of the compatible end seemed efficient, whereas the base pairings were not as stringent as the 5′-end phosphorylation; the TA type VOC was active with T, C, and G protrusions, whereas the blunt type reacted with all protrusions (Fig. 2B). Although the stringency of ligation can be enhanced by adding sodium chloride^30^, increased stringency was achieved at the risk of severe loss of ligation efficiency (not shown). The specificity of the VOCs for the unphosphorylated substrates seemed ideal for the second adaptor tagging after TACS ligation (see Introduction). Therefore, we proceeded to the development of a new library preparation protocol.

### The TACS-TOPO scheme can reduce adaptor dimer formation

**Figure 3:**
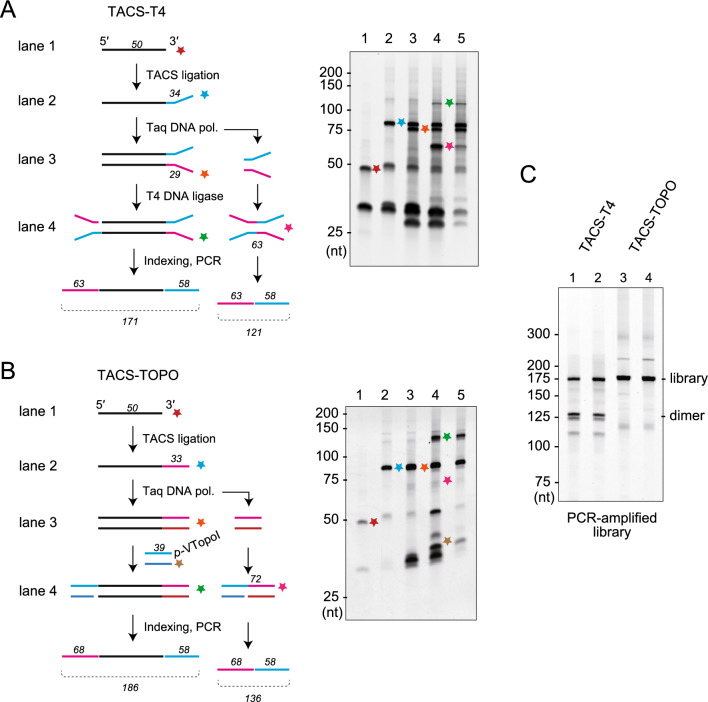
Comparison of library preparation schemes from single-stranded DNA. (**A**,**B**) TACS-T4 (**A**) and TACS-TOPO (**B**). The scheme (left) and analysis of DNA at each reaction step with denaturing gel electrophoresis (right) are shown. The numbers in italic fonts indicate the length of DNA used. The colored stars in the left scheme correspond to the bands in the right gel images. The lanes are labeled as follows: (1) before TACS ligation; (2) after TACS ligation; (3) after complementary strand synthesis with Taq DNA polymerase; (4) after second adaptor tagging; and (5) after SPRI purification. (**C**) A gel image showing the analysis of PCR-amplified libraries. DNA fragments amplified from libraries prepared using TACS-T4 (lanes 1 and 2: technical duplicates) and TACS-TOPO (lanes 3 and 4: technical duplicates) were loaded onto a denaturing gel. For both libraries, 20 cycles of PCR amplifications were conducted. Adaptor dimers were only evident in the libraries prepared with TACS-T4. The original gel images are provided in Supplementary Figure S7.

As VOC showed efficient adaptor tagging of the 5′-unphosphorylated dsDNA and did not react with 5′-phosphorylated DNA (Fig. 2B), we considered that VOC could realize efficient second adaptor tagging without adaptor dimer formation. When using 83 ng (5 pmol) of random 50-mer (N50) as a model target, the yield of the library with two adaptor sequences attached was low with the TACS-T4 scheme (Fig. 3A, lane 4, green star). On the contrary, a drastically improved library yield was observed with the TACS-TOPO scheme (Fig. 3B, lane 4, green star). Further, the PCR-amplified libraries prepared using TACS-T4 contained a large quantity of adaptor dimer, whereas only an insert positive library was observed with the TACS-TOPO scheme (Fig. 3C). These results indicated that the TACS-TOPO scheme is superior to the TACS-T4 scheme for high library yields and low adaptor dimer formation.

### Optimization of the TACS-TOPO scheme for highly sensitive, dimer-free library preparation from ssDNA

**Figure 4:**
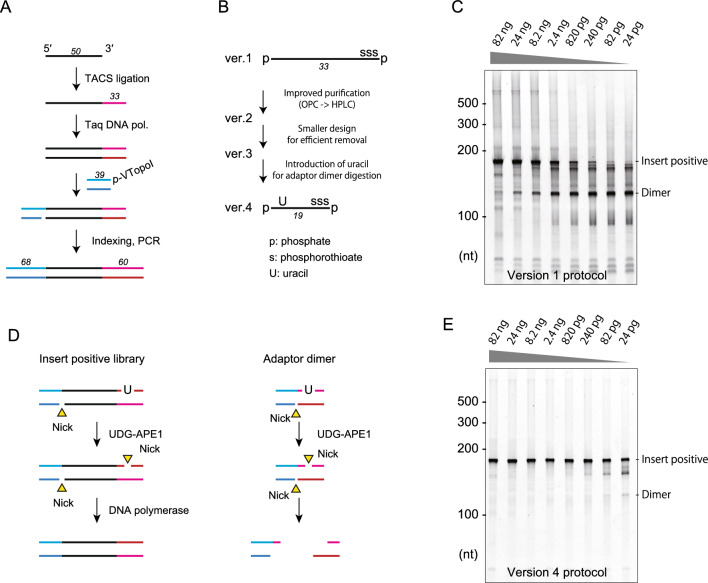
Improvements to the TACS-TOPO scheme. (**A**) The TACS-TOPO scheme. (**B**) Improvements applied to different versions of the protocol. OPC: oligonucleotide purification cartridge grade, HPLC: high-performance liquid chromatography purification grade. (**C**) The first version (ver. 1) produced several adaptor dimers. (**D**) The adaptor dimer degradation scheme introduced in the ver. 4 protocol. The uracil residue in the adaptor is degraded using UDG and APE 1 before PCR amplification of the library. This treatment causes the degradation of the adaptor dimer. (**E**) Library preparation using the TACS-TOPO ver. 4 protocol. The sensitivity of dimer-less library preparation was improved to realize highly sensitive library preparations from ssDNA. The original gel images for C and E are provided in Supplementary Figure S8.

Next, we investigated the sensitivity of adaptor dimer-free library preparation using the TACS-TOPO scheme. In this experiment, we again used N50 as a model and investigated the appearance of adaptor dimers (ver. 1, Fig. 4A,B and Supplementary Figure S2A). As shown in Fig. 4C, non-negligible amounts of adaptor dimers were observed when the input DNA was reduced to less than 24 ng. The imperfection of 5′-phosphorylation of the adaptor used in TACS ligation could explain this phenomenon, as we observed reduced adaptor dimer formation upon using an adaptor oligonucleotide with a higher purification grade (ver. 2, Fig. 4B and Supplementary Figure S2A and S2C). However, as further purification of the adaptor could not improve dimer formation (not shown), we needed to combine other experimental principles to reduce the adaptor dimers. The first was using a shorter adaptor to remove the dimers more efficiently (ver. 3, Fig. 4B, Supplementary Figure S2A and S2D). The other was adding a uracil residue near the 5′-side of the adaptor used for TACS-ligation to degrade the dimer before amplifying the library by PCR (ver. 4, Fig. 4B,D and Supplementary Figure S2A,E). Combined with the TACS-TOPO scheme, these two approaches resulted in dimer-free library preparations from 24 pg of N50 (Fig. 4E and Supplementary Figure S2E,G), corresponding to the DNA amount from four human cells. Therefore, adaptor dimer-free library preparation from small amounts of ssDNA has become practical using TACS-TOPO ver. 4.

### Library preparation from short single-stranded cell-free DNA in peripheral blood

**Figure 5:**
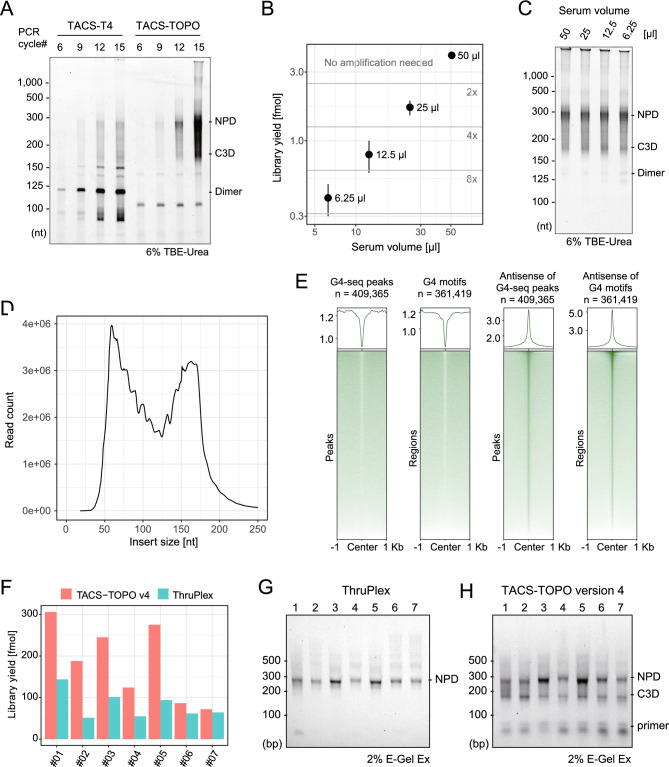
Sequencing library preparation from cfDNA. (**A**) Comparison of libraries prepared using TACS-T4 and TACS-TOPO. (**B**) Library yields of TACS-TOPO ver. 4 from various volumes of serum are shown. The zones separated with horizontal lines indicate the magnitude of amplification needed for sequencing to obtain 90 Gb reads. (**C**) Gel image showing the analysis of amplified cfDNA libraries prepared using TACS-TOPO ver. 4. After denaturing gel electrophoresis on a 6% gel, the SYBR gold-stained gel was photographed. (**D**) Size distribution of the reads mapped to the human reference genome. A library prepared from 50 µL serum using TACS-TOPO ver. 4 was sequenced and mapped to the human reference genome. (**E**) Colocalization of mapped reads on G4-seq peaks and G4 motifs. From the mapped reads, fragments of 35–75 nt were selected and analyzed for their colocalization with the G4-seq peaks and G4 motifs. The same serum (No. 1 in Supplementary Table S2) was used for (**A**–**E**). (**F**–**H**) Sequencing libraries prepared from 50 µL serum or plasma obtained from several sources are shown (Supplementary Table S2). The library yields (**F**) and gel electrophoresis images of the amplified libraries are shown for the ThruPlex DNA seq kit (**G**) and TACS-TOPO ver. 4 (**H**). The 6% TBE-Urea at the bottom of the gel image indicates analysis on denaturing gel electrophoresis using 6% Novex TBE-Urea gel (Thermofisher Scientific), and 2% E-Gel Ex is indicative of analysis on E-Gel Ex (2%) (Thermofisher Scientific). The original gel images for A, C, G, and H are provided in Supplementary Figure S9.

Previously, we identified a number of 50-nucleotide-long, single-stranded cfDNAs in the cell-free fraction of human peripheral blood and named it C3D^10^. We applied the TACS-T4 scheme to analyze cfDNA and found that the C3D comprises a new class of cfDNA. However, dominated amplification of the adaptor dimer using the TACS-T4 scheme required labor-intensive removal of the dimer molecule by repeating the SPRI purification steps^10^. Because the optimized TACS-TOPO scheme is effective for dimer-less library preparation from a limited amount of ssDNA (Fig. 4E), we applied TACS-TOPO ver. 4 for library preparation from cfDNA. For this analysis, we used a pooled human serum, which was obtained from a commercial source, as a model (Supplementary Table S2, entry number 1). As shown in Fig. 5A, sequencing libraries were prepared without adaptor dimer formation using TACS-TOPO ver. 4.

In contrast, a high rate of adaptor dimer formation was observed with TACS-T4 even after performing an SPRI-based purification for dimer removal (Fig. 5A). The appearance of two previously identified major peaks corresponding to nucleosome-sized cfDNA (nucleosome-protected DNA, or NPD; around 160-nt-long inserts) and C3D in the PCR-amplified libraries supported the feasibility of TACS-TOPO ver. 4 for cfDNA analysis (Fig. 5A). We found that cfDNA extracted from 50 µL serum was sufficient for amplification-free sequencing library preparation and that sequencing libraries could be successfully prepared from 6.25 µL of serum with three cycles of PCR (eightfold amplification, Fig. 5B,C). These results indicate that TACS-TOPO greatly enhanced the sensitivity of cfDNA analysis because TACS-T4 required at least 250 µL of plasma or serum and 10 cycles of PCR amplification^10^. We sequenced the TACS-TOPO library prepared from 50 µL of human serum to find two major peaks in the size distribution of mapped read alignments (Fig. 5D).

C3D was defined as cfDNA fragments ranging 35–75 nt in length. The mapped reads of C3D are known to form sharp peaks, a representative characteristic of C3D (Supplementary Figure S3)^10^. The C3D peaks colocalize with several genomic features, and the antisense of G-quadruplex (G4) structures is best colocalized with the C3D peaks^10^. Therefore, we investigated whether the C3D peaks called with the reads obtained with the TACS-TOPO ver. 4 colocalized with the G4 structures. The C3D peaks were well colocalized with the antisense strands of G4 motifs and peaks of G4-seq (Fig. 5E and Supplementary Figure S3). These results indicate that TACS-TOPO can capture the previously known features in cfDNA analysis.

To compare the differences between TACS-TOPO ver. 4 with a conventional dsDNA-adapted library preparation protocol, we investigated the yields and size distribution of the sequencing libraries prepared from the blood cfDNA extracted from plasmas and sera of healthy individuals (for details, see Supplementary Table S2). For this comparison, we chose the ThruPlex DNA seq kit from Takara Bio as it is a standard kit with excellent library yields. As shown in Fig. 5F, the TACS-TOPO ver. 4 consistently showed superior library yields to the ThruPlex kit. A distinctive difference between the two protocols was observed for the size distribution of the amplified library; only one major peak was observed with the ThruPlex kit (Fig. 5G), whereas two peaks were evident with the TACS-TOPO ver. 4 protocol (Fig. 5H). Since the molar concentration of C3D in the blood is comparable to that of NPD^10^, the ssDNA-adapted library preparation protocol is expected to produce a higher library yield than the conventional dsDNA-specific protocols. Therefore, considering the high efficiency, the higher library yields observed with TACS-TOPO ver. 4 compared to those observed with ThruPlex are quite reasonable.

### TACS-TOPO scheme realizes highly sensitive library preparation from ancient DNA

Owing to an advanced state of degradation, most DNA extracted from ancient samples is fragmented and single-stranded. Therefore, library preparation methods for such samples should be adapted for ssDNA. Accordingly, the usefulness of ssDNA-adapted library preparation for ancient DNA was demonstrated by Meyer et al.^5^ and Gansauge et al.^6,7^. Moreover, we previously showed that the TACS-T4 scheme greatly improved library yields compared to the methods established by Gansauge et al.^6,7^ when applied to cfDNA library preparation^10^. Furthermore, the TACS-TOPO scheme performed better than the TACS-T4 scheme in cfDNA library preparation (Fig. 5A). Therefore, the TACS-TOPO scheme should be more suitable for library preparation from ancient samples, and thus, we subsequently performed ancient DNA analysis using the TACS-TOPO scheme.

We chose DNA extracted from human bones excavated from the Inome cave site (Inome, Supplementary Table S3)^24^ and the Shimekake site (Shime and Shime-t, Supplementary Table S3)^25^ as model samples. As these sites are of the Tumulus and late Jomon eras, respectively and these bones were of individuals who lived 1000–3000 years ago (Supplementary Table S3)^31–33^, the extracted DNAs were expected to be heavily degraded and damaged. In addition to the degradation, ancient DNA contains large amounts of deaminated cytosine residues or uracil. Because the ThruPlex kit is unsuitable for uracil-containing ancient DNA, we used the KAPA Hyper Prep kit combined with KAPA HiFi HotStart Uracil Ready Mix as a control protocol adapted only for dsDNA.

**Figure 6:**
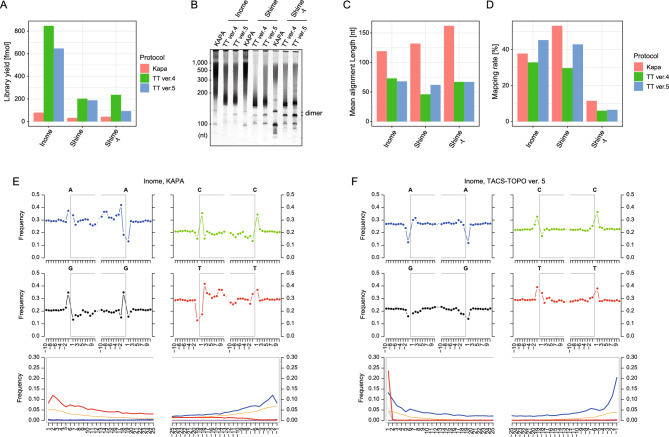
Comparison of library preparation from ancient DNA using TACS-TOPO and a conventional protocol adapted only for dsDNA (KAPA Hyper Prep kit). (**A**–**D**) Comparisons of library yields after 12 cycles of PCR amplification (**A**), gel electrophoresis of amplified sequence libraries (**B**), mean alignment length of sequenced reads (**C**), and mean mapping rates of the reads on the human reference genome (**D**) are shown. The KAPA Hyper Prep kit was chosen as a representative protocol for its superior sensitivity among commercial library preparation kits and its tolerance for uracil-containing DNA. E and F. Mutation patterns were drawn using mapDamage^28,29^ for libraries prepared using the KAPA Hyper Prep kit (**E**) and TACS-TOPO ver. 5 (**F**). The four upper plots for each library show the base frequency outside and inside the read. The open grey box in the plots corresponds to the read. The bottom plots are the base substitution frequencies at relative positions from the 5′-(left) and 3′-(right) ends of the reads. The frequencies of C to T (red), G to A (blue), all other substitutions (gray), and soft-clipped bases (orange) are shown. Kapa: KAPA Hyper Prep kit; TT ver. 5: TACS-TOPO ver. 5. The original gel images for B are provided in Supplementary Figure S10.

As expected, the yields of the libraries were 5.4–10.7 times higher with the TACS-TOPO ver. 4 than that with the KAPA Hyper Prep kit (Fig. 6A). The library insert was smaller with TACS-TOPO ver. 4 (two peaks at 50 nt and 400 nt) than that with the KAPA Hyper Prep kit (one peak at more than 200 bp) (Fig. 6B), and the mean insert sizes determined using small-scale sequencing reflected the distribution patterns of the libraries (Fig. 6C). The mapping rates to the human reference genome were almost the same between the TACS-TOPO ver. 4 and KAPA Hyper Prep kits (Fig. 6D). These results confirmed the high degree of degradation of ancient DNAs into short ssDNA and the superiority of ssDNA-adapted library preparation protocol for analyzing such samples.

In ancient DNA, deamination events increase toward the ends of the DNA molecules, so the frequencies of C to T conversions in sequenced reads tend to increase at the ends of DNA. This change in conversion frequency is a well-used indicator of ancient DNA, and is clearly observed with the KAPA Hyper Prep kit (Fig. 6E and Supplementary Figure S4A,D,G). In contrast, with the TACS-TOPO ver. 4 protocol, we observed such mutation patterns only on the 5′-side of the reads (Supplementary Figure S4B,E,H). We realized that the uracil DNA glycosidase treatment to degrade adaptor dimers in the TACS-TOPO ver. 4 protocol caused the disappearance of the C to T converted reads at the 3′-end. Therefore, we investigated alternative modifications that could substitute the uracil residue for the adaptor dimer removal. We found that the AP site analog dSpacer (dSp) could be used for this purpose. As dSp is known to be cut by APE 1, we initially used APE 1 before PCR amplification. However, for unknown reasons, APE 1 is unnecessary for repressing adaptor dimer formation (not shown). We defined this dSp-based protocol as TACS-TOPO ver. 5 (Supplementary Figure S2A,F,G) and applied it to analyze the ancient DNA again.

With the TACS-TOPO ver. 5, similar improvements as those with the TACS-TOPO ver. 4 were observed for library yields (Fig. 6A), insert sizes (Fig. 6B,C), and mapping rates (Fig. 6D). On the contrary, the base-substitution patterns of reads obtained with the TACS-TOPO ver. 5 differed from those of TACS-TOPO ver. 4 (Supplementary Figure S4B,E,H). Closer to the reads' ends, higher mutation rates were observed at both ends of the reads with the TACS-TOPO ver. 5 (Fig. 6F, Supplementary Figure S4C,F,I). Interestingly, opposite mutation patterns were observed between the TACS-TOPO ver. 5 and the KAPA Hyper Prep kit. With the TACS-TOPO ver. 5, C to T mutations increased at both ends of the reads (Fig. 6F, Supplementary Figure S4C,F,I). However, with the KAPA Hyper Prep kit, C to T mutations increased only at the 5′-terminal of fragments, whereas G to A mutations were observed in the 3′-terminal of fragments (Fig. 6E, Supplementary Figure S4A,D,G). This is a typical difference between ssDNA-adapted protocols and protocols adapted only for dsDNA^5^.

## Discussion

In the present study, intending to establish an efficient and dimer-less procedure for library preparation from short ssDNA, we investigated the use of VTopoI for second adaptor tagging after TACS ligation. While the VOC is specific to the unphosphorylated 5′-end of DNA (Fig. 2) and the use of the VOC effectively suppresses adaptor dimer formation, the dimer was still observed when the sample amount was limited (Fig. 4C). Thus, we combined a uracil-containing adaptor and a UDG treatment for dimer removal. The resultant TACS-TOPO ver. 4 was effective for adaptor-dimer-free library preparation from limited sample input (Fig. 4D) and was successfully applied to the library preparation from human blood cfDNA (Fig. 5). As the TACS-TOPO could efficiently convert short ssDNA that exist in the cell-free fraction of blood into sequencing libraries, the libraries prepared from blood cfDNA displayed different compositions from the ones prepared using conventional protocols only adapted for dsDNA (Fig. 5F–H). We also showed that the TACS-TOPO could greatly improve the yields of sequencing library preparation from ancient DNA (Fig. 6A). These successful examples support the practical usability of the TACS-TOPO scheme.

### VTopoI realizes a practical protocol for sequencing library preparation from short ssDNA

Using the VOC for the second adaptor tagging resulted in enhanced adaptor tagging and reduced adaptor dimer formation (Fig. 3), and the employment of VOC can bring additional practical benefits. The first is its reaction speed; only 15 min was sufficient to complete the second adaptor tagging at room temperature (Fig. 3 and Supplementary Methods). The VTopoI-based cloning reaction is quick and can be completed in less than 5 min^21^; therefore, the TACS-TOPO scheme may be shortened with further optimization. Another benefit of VOC is its operational simplicity. Since VOC requires no additional materials, adding only VOC to the reaction is sufficient to start the reaction. Therefore, VOC achieves a short reaction time and operational simplicity in addition to excellent library yields and reduced adaptor dimer formation. These benefits would greatly improve the practical usability of sequence library preparation from ssDNA.

### The adaptor dimer formation is still a hurdle for sequencing library preparation from short ssDNA

Although the use of VOC for second adaptor tagging in the TACS-TOPO scheme effectively reduces adaptor dimer formation (Fig. 3C), adaptor dimer formation was still detected with decreased input DNA (Fig. 4B). This observation is seemingly paradoxical because VOC would not react with a 5′-phosphorylated adaptor (Fig. 2B). We think that 5′-unphosphorylated oligonucleotide contamination in the 5′-phosphorylated adaptor caused this phenomenon because less adaptor dimer formation was observed with a higher purification grade. In the current study, the 5′-phosphorylation of the adaptor was chemically attached. Since the chemical reaction and product purification are difficult to perfect, contamination of unphosphorylated DNA in the 5′-phosphorylated adaptor is unavoidable. If this explanation is true, constant production of adaptor dimer is expected if a fixed amount of adaptor is used for the first adaptor tagging of ssDNA.

The reduction and degradation of the adaptor dimer are the two potential solutions for this shortcoming. The TACS-TOPO ver. 4, which degrades the adaptor dimer with UDG, greatly repressed the amplification of the adaptor dimer and realized a highly sensitive dimer-free library preparation from ssDNA (Fig. 4C,D). However, the application of UDG-based degradation is sometimes difficult for uracil-containing samples; therefore, the TACS-TOPO ver. 4 could not be used as a general procedure for sequencing library preparation from ssDNA. In contrast, TACS-TOPO ver. 5, which includes a dSp in the adaptor used with TACS ligation, is useful for reduced adaptor dimer formation. The TACS-TOPO ver. 5 could be used for any ssDNA but is less sensitive than the TACS-TOPO ver. 4 regarding dimer-less library preparation (Supplementary Figure S2). Thus, further studies are required to establish a general and sensitive procedure for dimer-free library preparation from ssDNA.

### The TACS-TOPO scheme provides a general solution for efficient and sensitive library preparation from ssDNA

In the current study, we focused on library preparation from ssDNA, especially from short strands in the cell-free fraction of the blood and from ancient DNA. Because the target DNA is too short to be separated from the adaptor dimer via a size-dependent DNA purification procedure, we needed different principles, such as dU and dSp-based adaptor dimer removal (i.e., ver. 4 and ver. 5 protocols). However, if ssDNA is long enough to be separated from the adaptor dimer via size fractionation, the ver. 3 protocol can be used. As described in Materials and Methods, after immobilizing the adaptor-tagged target DNA on the SPRI beads, the magnetic beads were washed with a solution containing 32% PEG400 to remove the unreacted adaptor molecules. This condition was effective in removing the unreacted adaptor and primer, but not the adaptor dimer. Indeed, we could prepare a sequencing library from large target ssDNA without using uracil or dSp-containing adaptors while enforcing stringent size fractionation conditions (not shown). As such, with appropriate options, the TACS-TOPO scheme can be used for sequencing library preparation from ssDNA; not only for short strands, but for longer ones as well.

### Supplementary Information

Supplementary Information.

Supplementary Tables.

## Data Availability

All sequence data obtained in this study were deposited to NCBI GEO and NCBI SRA under the accession numbers GSE222918 and PRJNA921947, respectively.
