# Supplementary material for: A highly efficient scheme for library preparation from single-stranded DNA
Source: Sci Rep. 2023 Aug 25;13:13913. doi: 10.1038/s41598-023-40890-3 (PMC10457334; doi:10.1038/s41598-023-40890-3)
Supplement: Supplementary file 1 — Supplementary Information. [file 41598_2023_40890_MOESM1_ESM.docx]

**Supplementary Information**

A highly efficient scheme for library preparation from single-stranded DNA

Fumihito Miura^1,*^, Hideaki Kanzawa-Kiriyama ^2^, Osamu Hisano^1,3^, Miki Miura^1^, Yukiko Shibata^1^, Noboru Adachi^4^, Tsuneo Kakuda^4^, Ken-ichi Shinoda^5^ and Takashi Ito^1^

^1^ Department of Biochemistry, Kyushu University Graduate School of Medical Sciences, 3-1-1 Maidashi, Higashi-Ku, Fukuoka 812-8582, Japan

^2^ Department of Anthropology, National Museum of Nature and Science, 4-1-1 Amakubo, Tsukuba, Ibaraki 305-0005, Japan

^3^ Department of Clinical Radiology, Kyushu University Graduate School of Medical Sciences, 3-1-1 Maidashi, Higashi-Ku, Fukuoka 812-8582, Japan

^4^ Department of Legal Medicine, Interdisciplinary Graduate School of Medicine and Engineering, University of Yamanashi, 1110 Shimokato, Chuo, Yamanashi 409-3898, Japan.

^5^ National Museum of Nature and Science, 4-1-1 Amakubo, Tsukuba, Ibaraki 305-0005, Japan

* To whom correspondence should be addressed:

Fumihito Miura

Tel: +81-92-642-6100

Fax: +81-92-642-6203

Email: fumihito@med.kyushu-u.ac.jp

**Contents**

Supplementary Figures S1~S12: Pages 2~16:

Supplementary Methods: Page 17

Supplementary Sequences: Page 18

Note: Supplementary Tables S1, S2, and S3 are provided in an Excel file.

**Supplementary Figures**


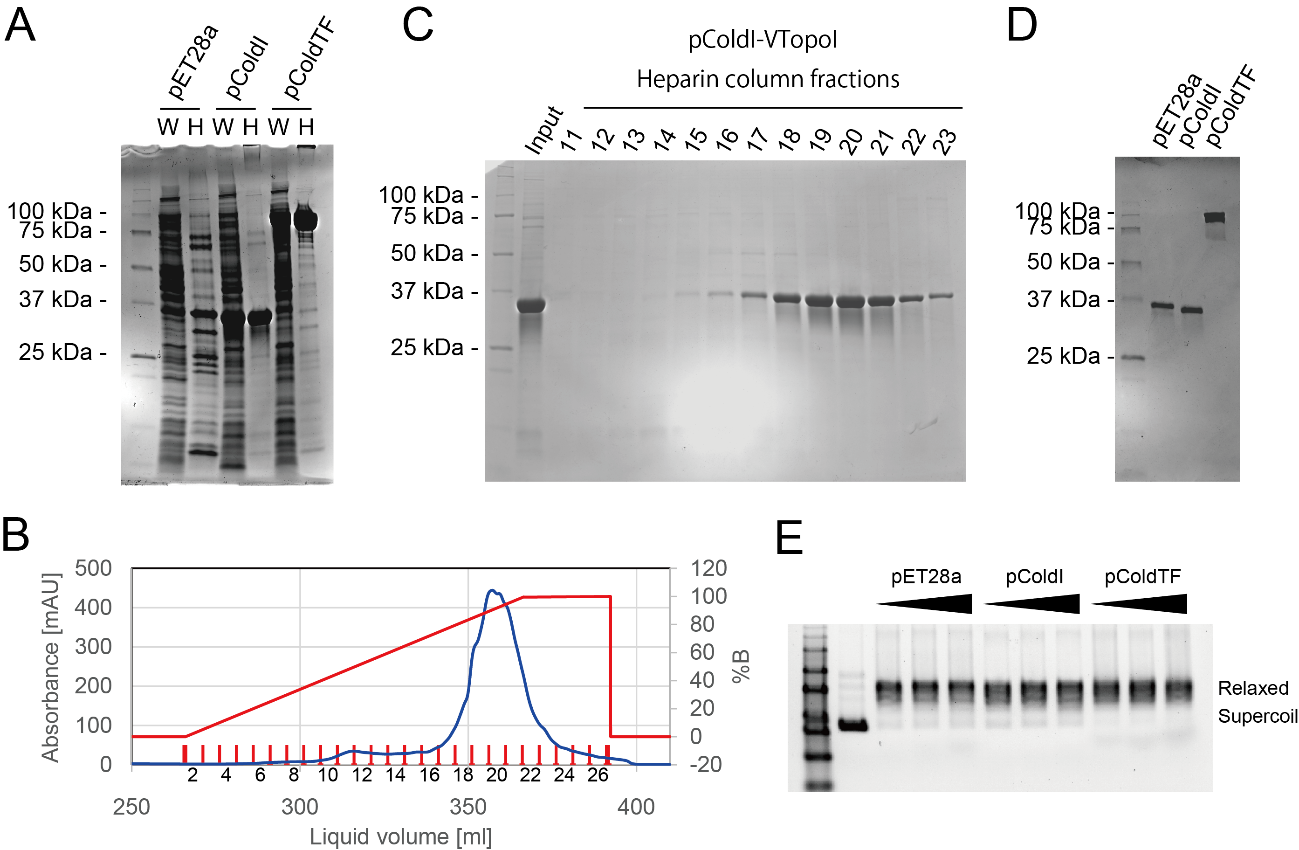


**Supplementary Figure S1**. Purification of Vaccinia virus topoisomerase I (VTopoI). A. Expression of VTopoI using three expression vectors. The expected protein sizes of VTopoI expressed with pET28a, pColdI, and pColdTF are 41.1 kDa, 40.1 kDa, and 91.0 kDa, respectively. B and C. Heparin column purification of VTopoI. An example of VTopoI expressed with pColdI. A chromatogram (B) and SDS-PAGE gel image (C) are shown. The blue and red lines in the chromatogram indicate the absorbance at 280 nm and the solution B content, respectively. The numbers on the gel image and the chromatogram indicate the fraction numbers. D. SDS-PAGE image for purified VTopoI proteins. E. Supercoil relaxation assays of purified VTopoI. For details, see Supplementary Methods. The original gel images for A, C, D, and E are provided in Supplementary Figure S11.


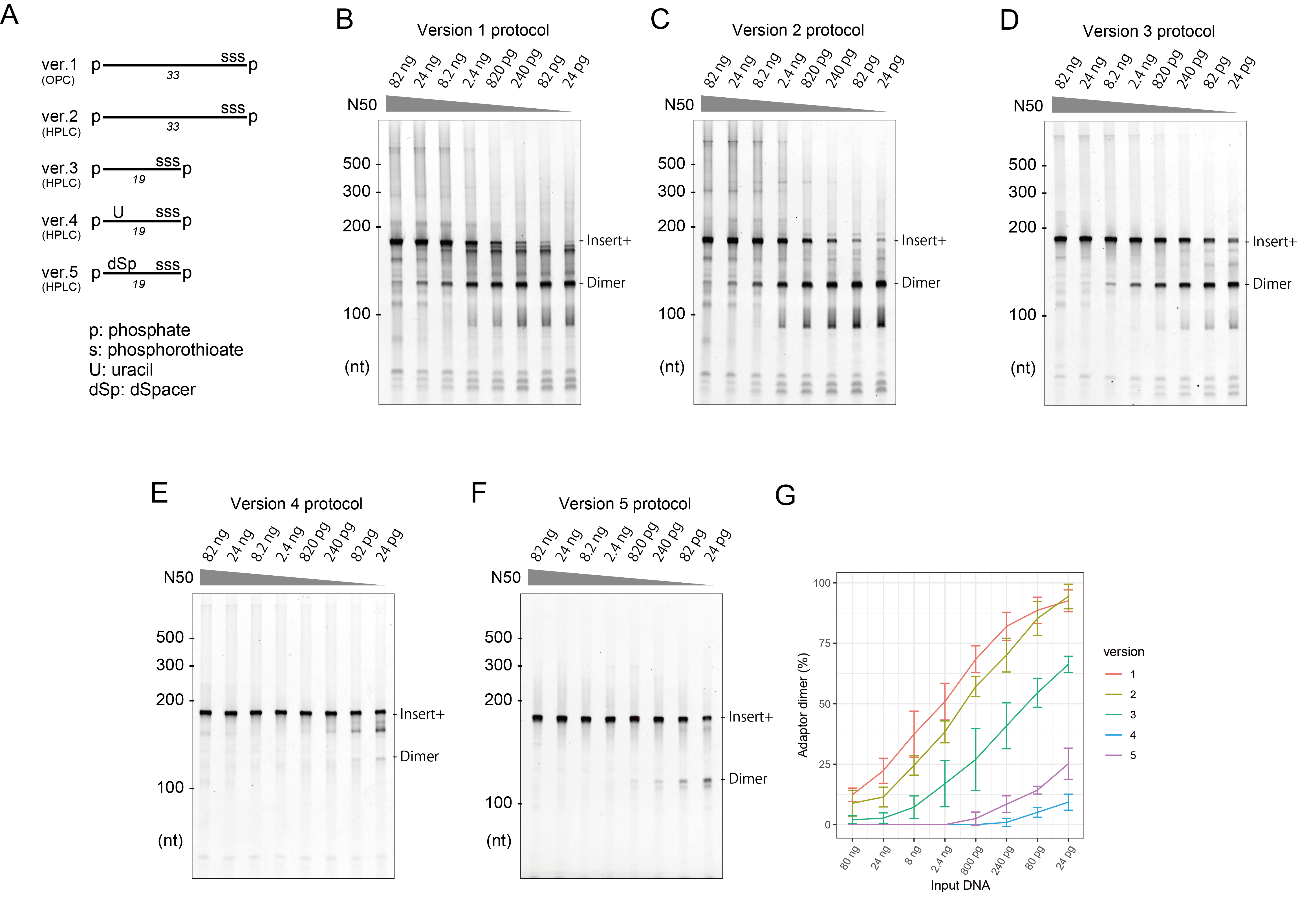


**Supplementary Figure S2**. Improvement of adaptors for dimer-less library preparations. A. The structures of the adaptors. B-G. TACS-TOPO protocols and dimer formation. Representative gel images of the PCR-amplified library (B-F) and summary of the relative amount of dimer formation (G). Libraries were prepared from various amounts of N50, PCR-amplified, and analyzed by denaturing gel electrophoresis. With improvements in the adaptor, the relative amounts of the dimer were reduced. For G, three independent experiments were performed. The original gel images for B-F are provided in Supplementary Figure S12.


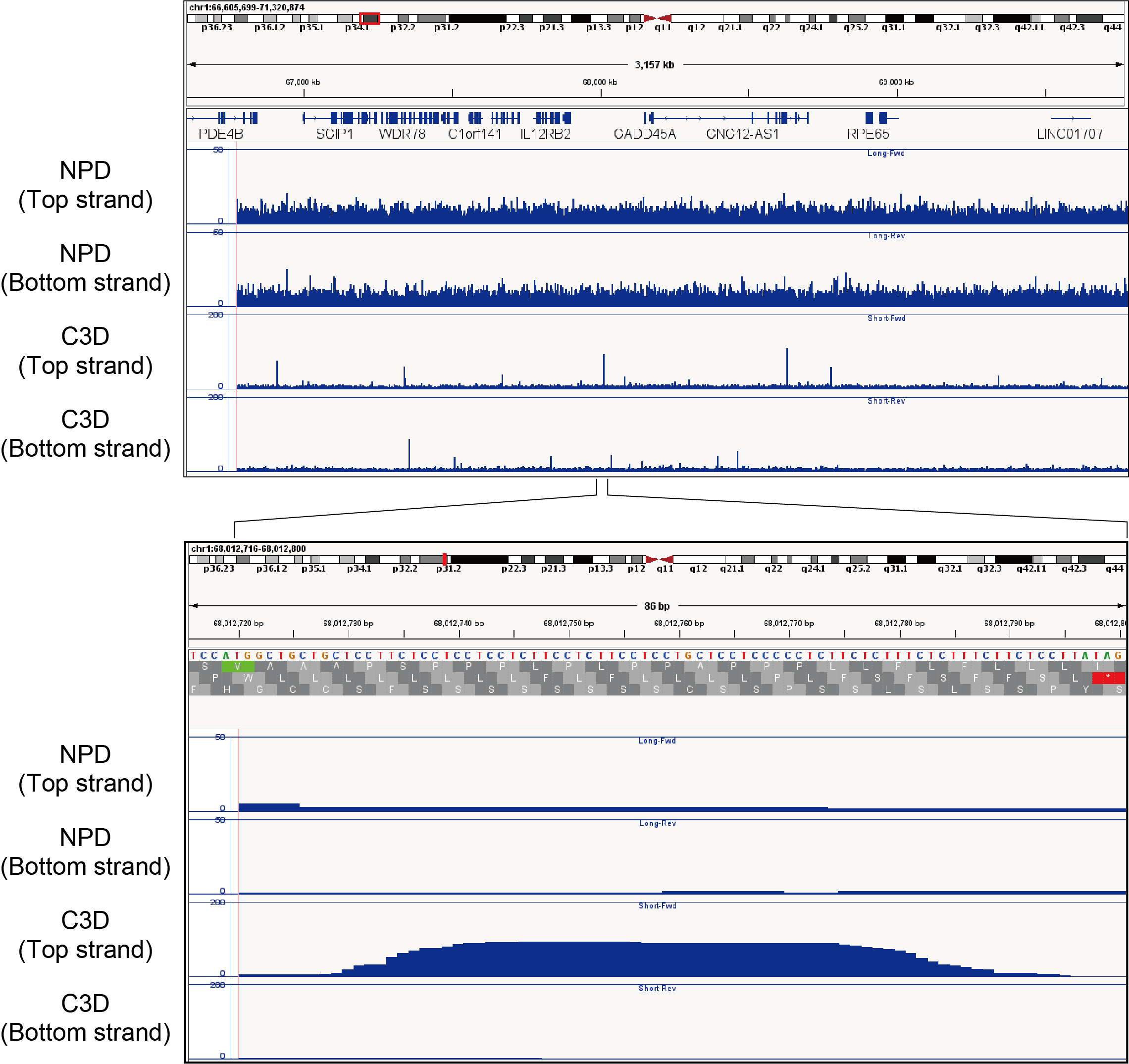


**Supplementary Figure S3**. A genome browser screenshot of representative C3D peaks. The mapped read coverages of NPD (cfDNA fragments of 147–190 nt in length) and C3D (35–75 nt in length) are shown for a randomly selected genomic locus (chr1, 66,605,699–71,320,874, top panel). The top and bottom strands are displayed separately. A typical C3D peak is enlarged (bottom panel).

*(Continues on the next page)*
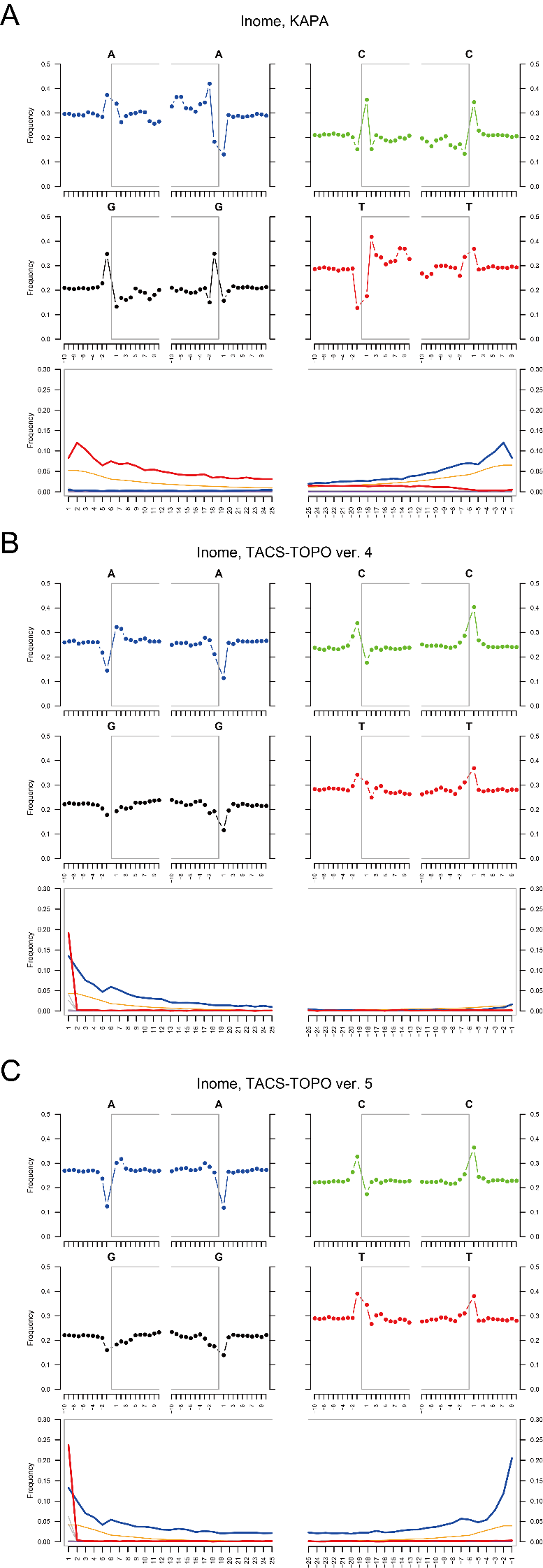


*
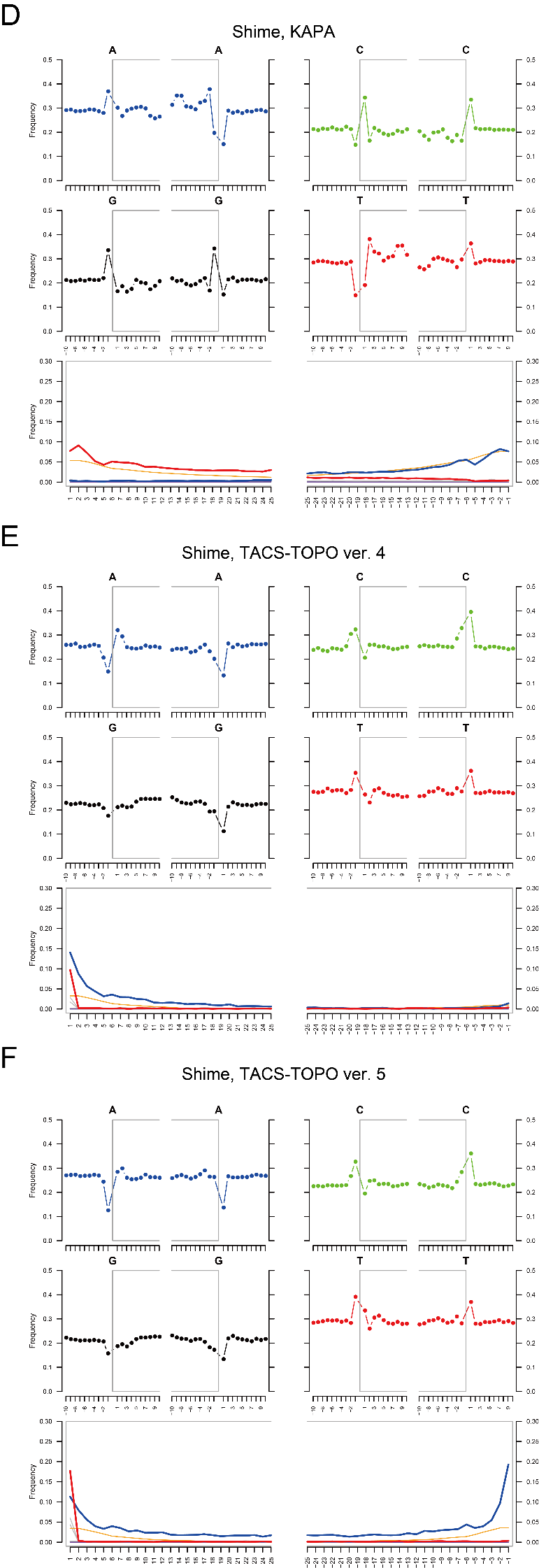
(Continues on the next page)*


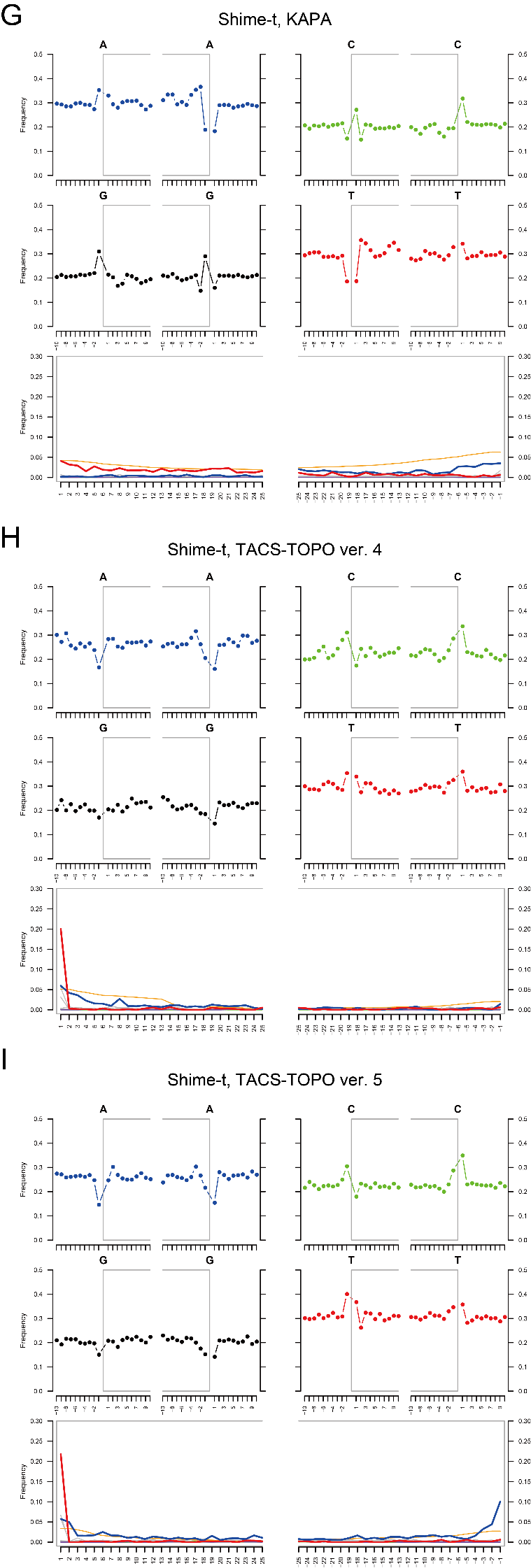


**Supplementary Figure S4**. Mutation patterns were drawn using mapDamage for libraries prepared with the KAPA Hyper Prep kit (A, D, and G), TACS-TOPO ver. 4 (B, E, and H), and TACS-TOPO ver. 5 (C, F, and I) for Inome (A–C), Shime (D–F), and Shime-t (G–I) (Supplementary Table S1). A and C are the same image presented in Figures 6E and 6F, respectively. The four upper plots for each library show the base frequency outside and in the read. The open grey box in the plots corresponds to the read. The bottom plots are the base substitution frequencies at relative positions from the 5′- (left) and 3′- (right) ends of the reads. The frequencies of C to T (red), G to A (blue), all other substitutions (gray), and soft-clipped bases (orange) are shown.


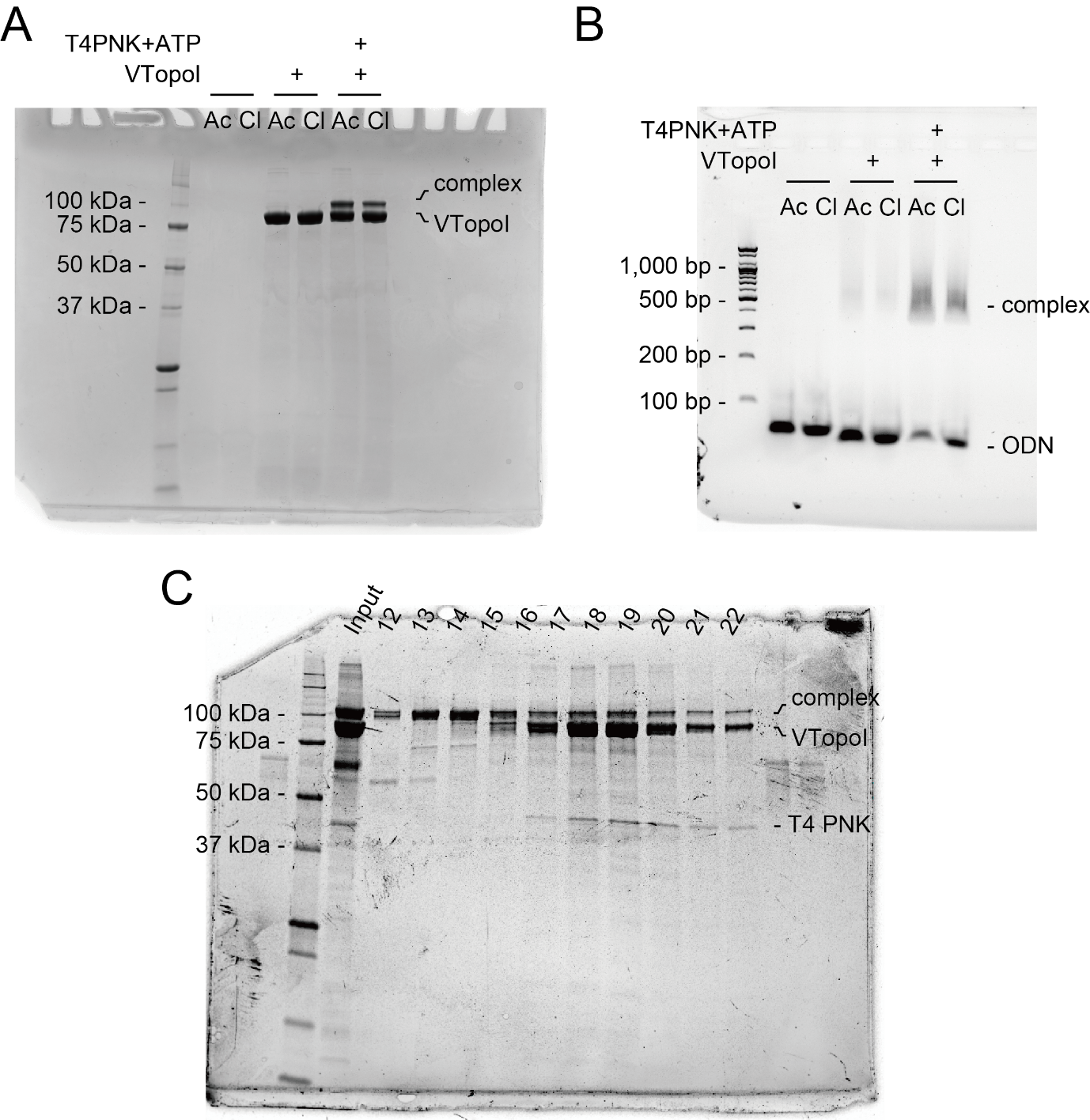


**Supplementary Figure S5**. **Formation and purification of the VTopoI-oligonucleotide complex (VOC) (The original gel images for Figure 1).** A. The original gel image for Figure 1B. B. The original gel image for Figure 1C. C. The original gel image for Figure 1D.


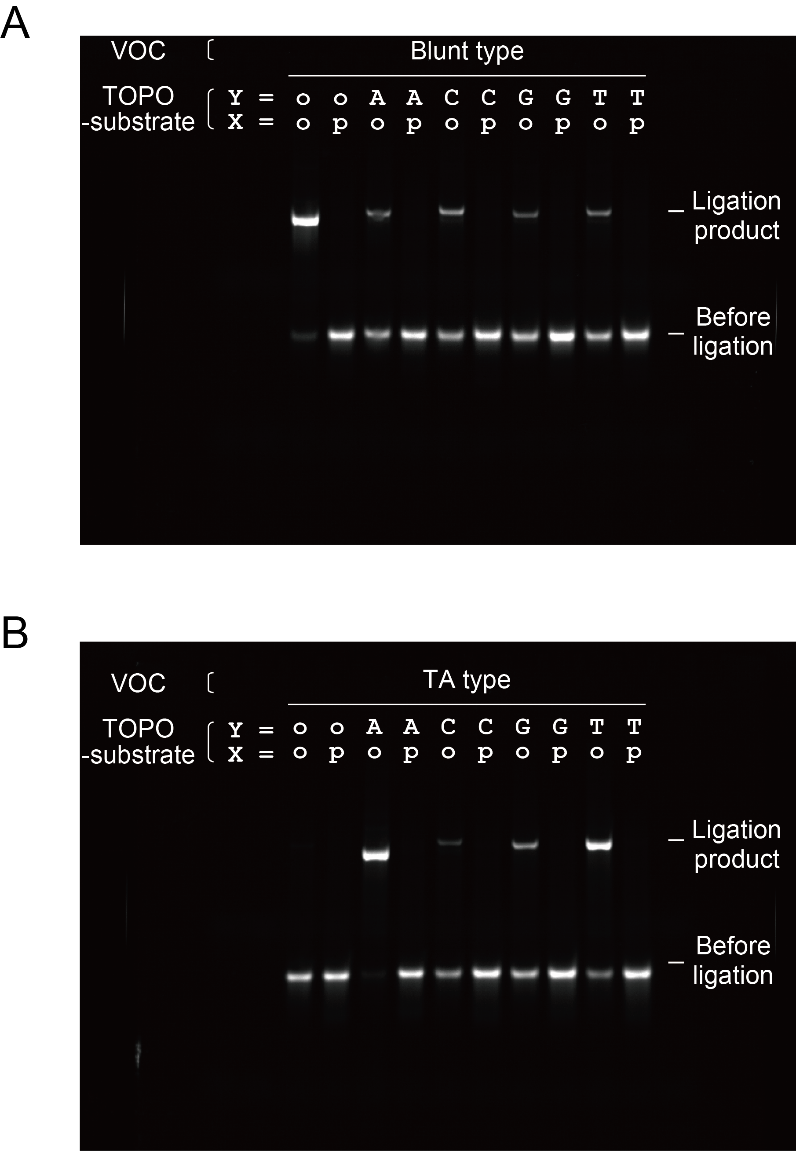


**Supplementary Figure S6**. **Substrate specificities of the VTopoI-oligonucleotide complex (VOC) (The original gel images for Figure 2).** A. The original gel image for Figure 2B (left panel). B. The original gel image for Figure 2B (right panel).


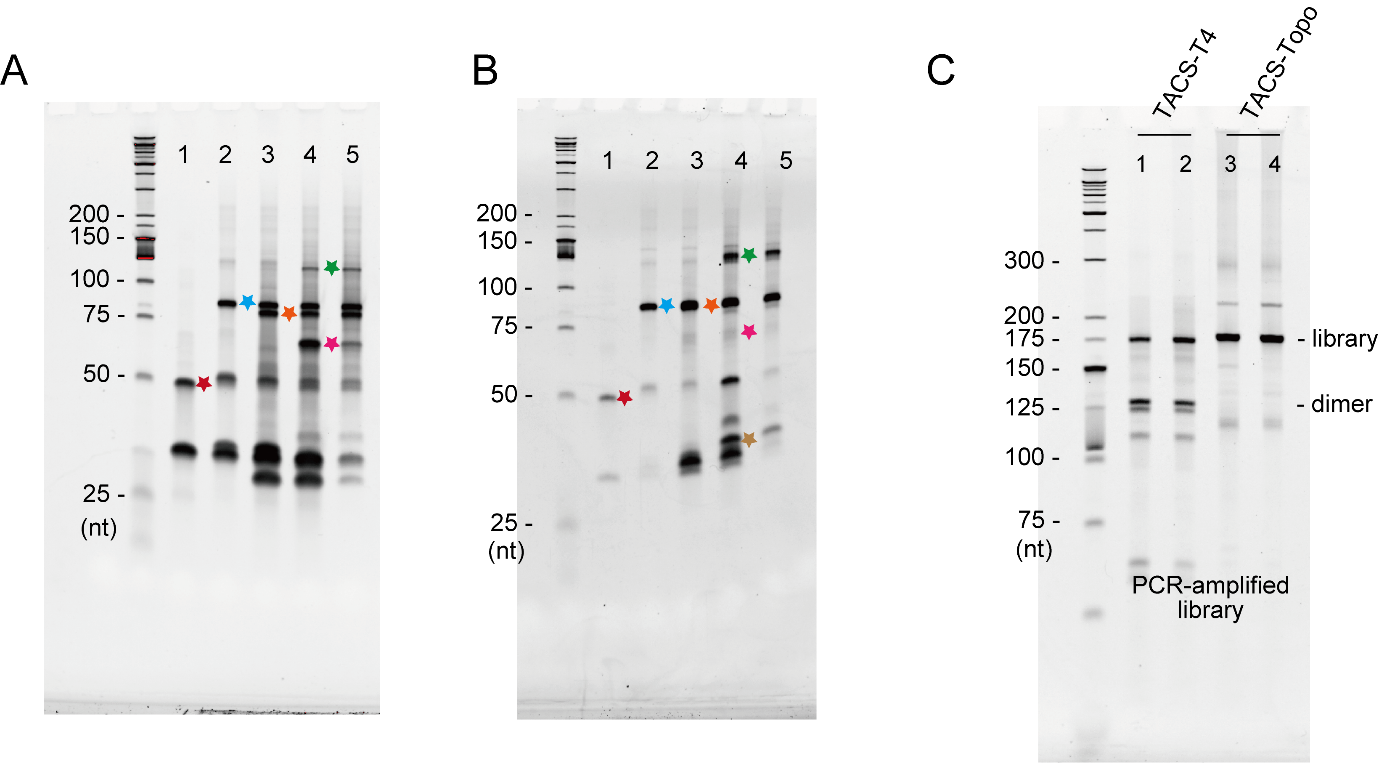


**Supplementary Figure S7**. **Comparison of library preparation schemes from single-stranded DNA (The original gel images for Figure 3).** A. The original gel image for Figure 3A. B. The original gel image for Figure 3B. C. The original gel image for Figure 3C.


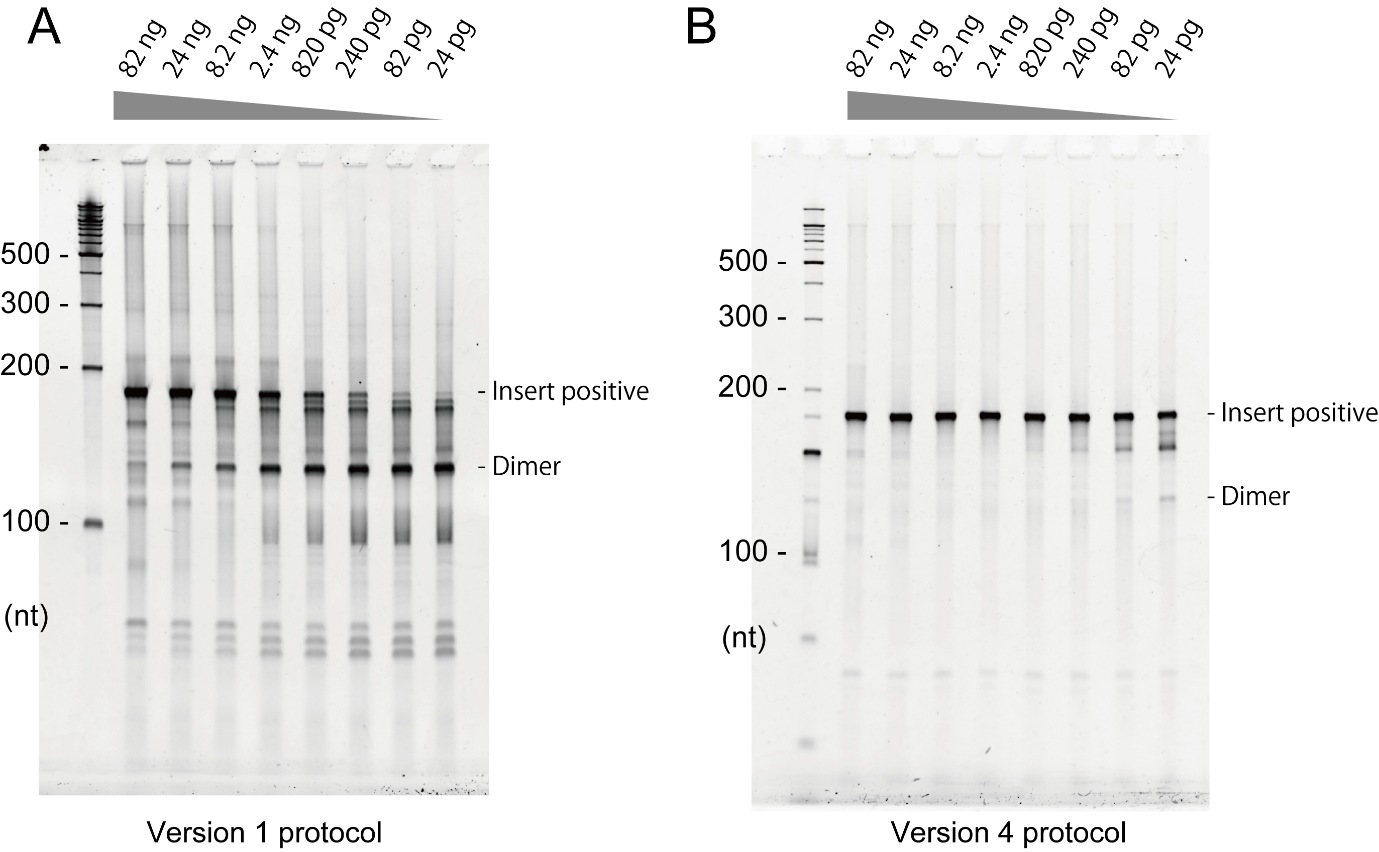


**Supplementary Figure S8**. **Improvements to the TACS-TOPO scheme (The original gel images for Figure 4).** A. The original gel image for Figure 4C. B. The original gel image for Figure 4E.


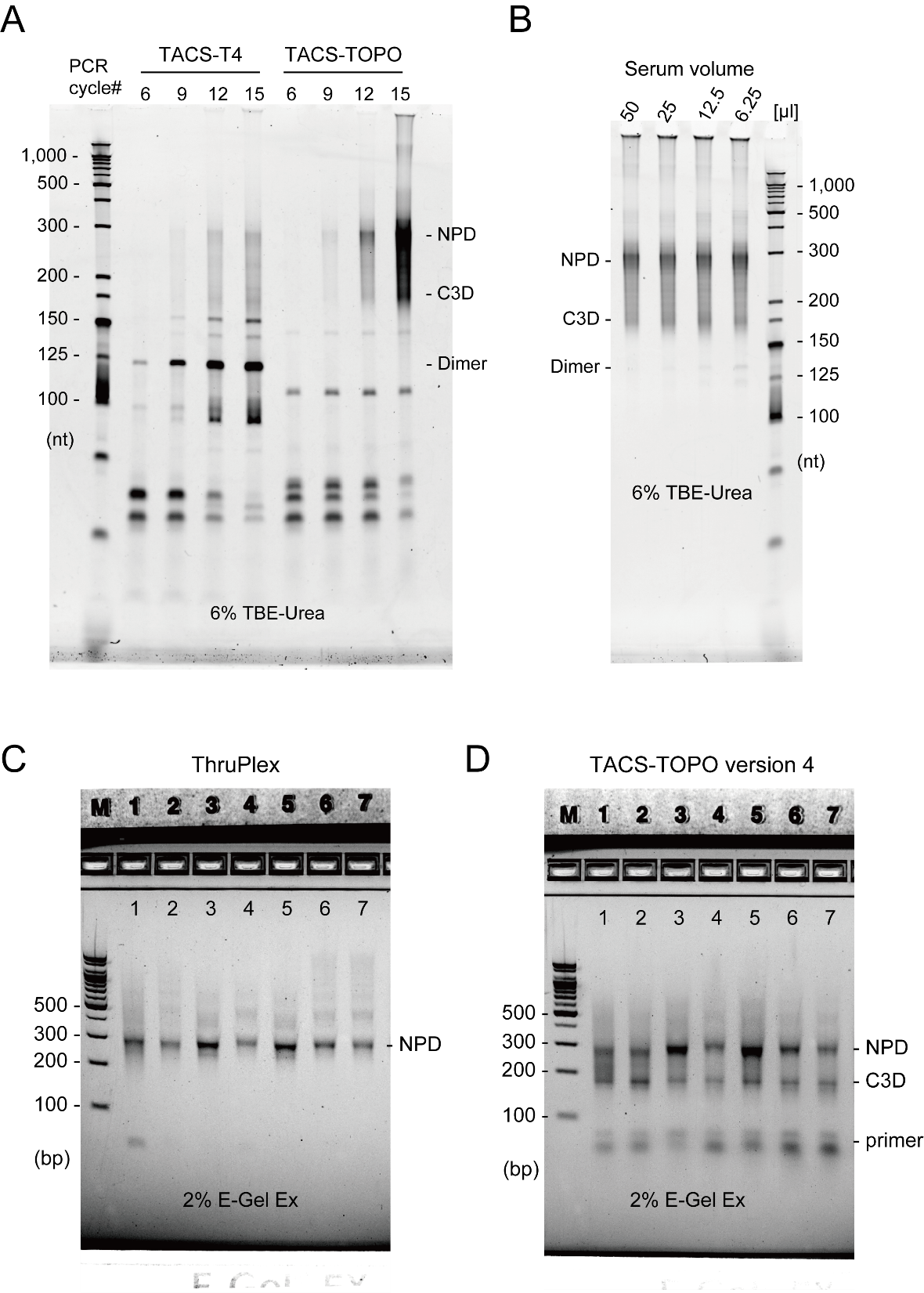


**Supplementary Figure S9**. **Sequencing library preparation from cfDNA (The original gel images for Figure 5).** A. The original gel image for Figure 5A. B. The original gel image for Figure 5C. C. The original gel image for Figure 5G. D. The original gel image for Figure 5H.


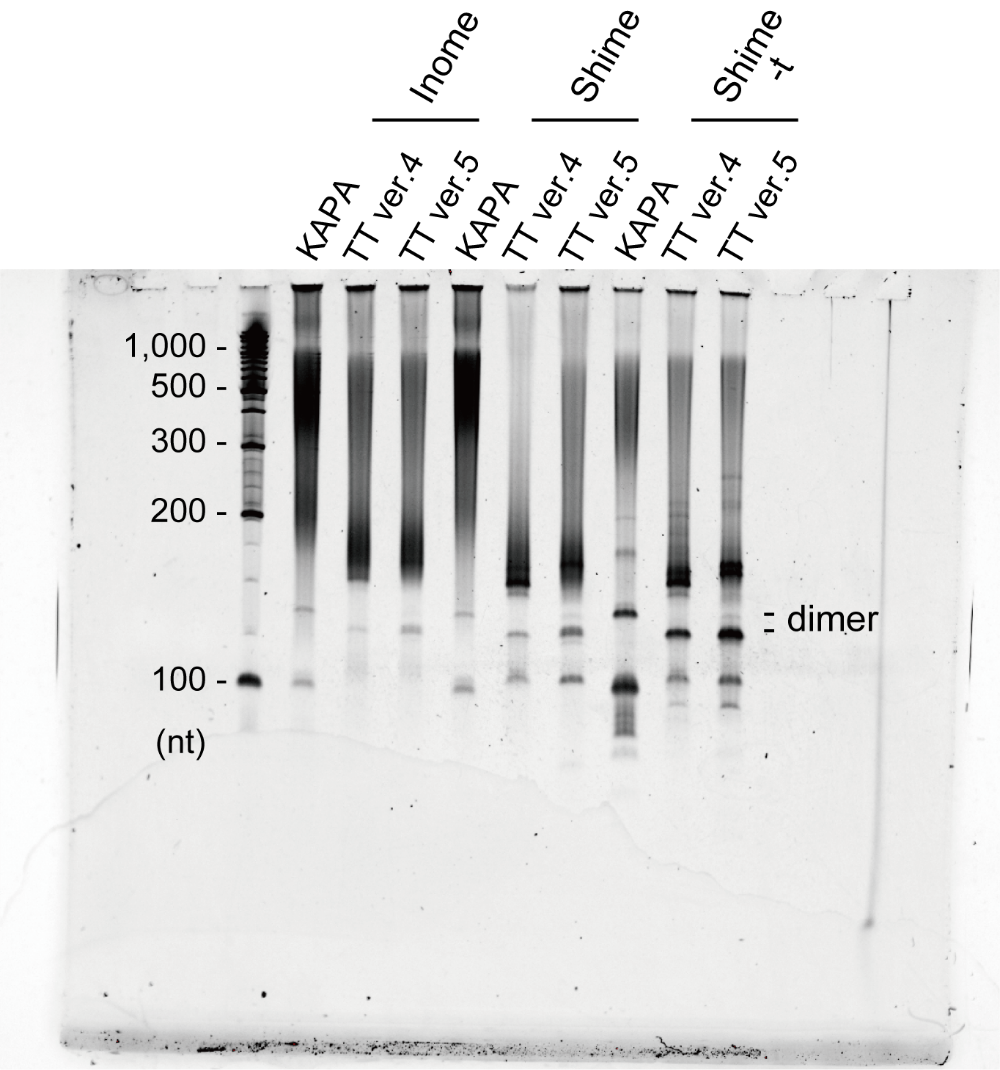


**Supplementary Figure S10**. **Comparison of library preparation from ancient DNA using TACS-TOPO and a conventional protocol adapted only for dsDNA (KAPA Hyper Prep kit) (The original gel image for Figure 6B).**


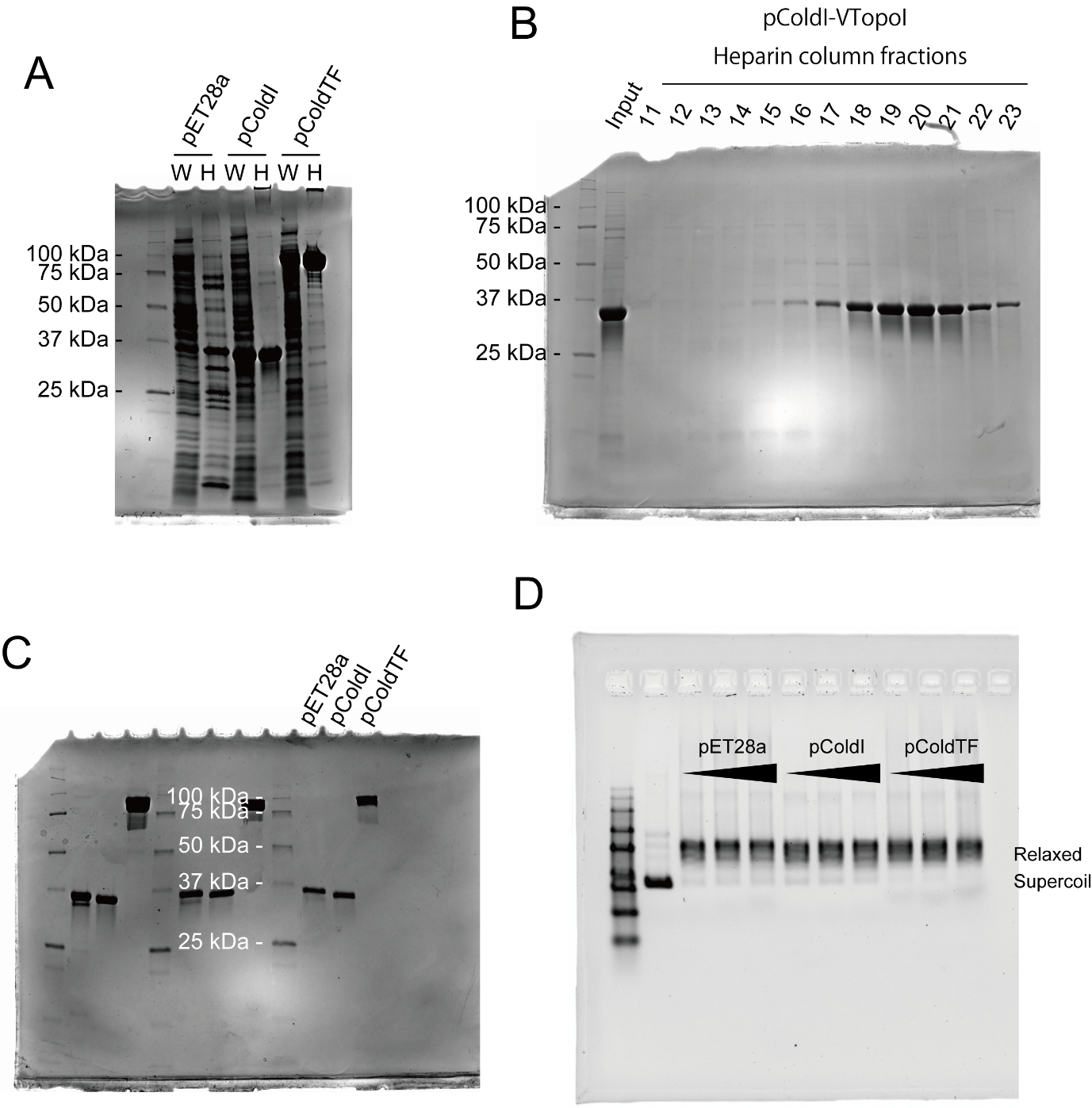


**Supplementary Figure S11**. **Purification of Vaccinia virus topoisomerase I (VTopoI) (The original gel images for Supplementary Figure S1).** A. The original gel image for Supplementary Figure S1A. B. The original gel image for Supplementary Figure S1C. C. The original gel image for Supplementary Figure S1D. D. The original gel image for Supplementary Figure S1E.


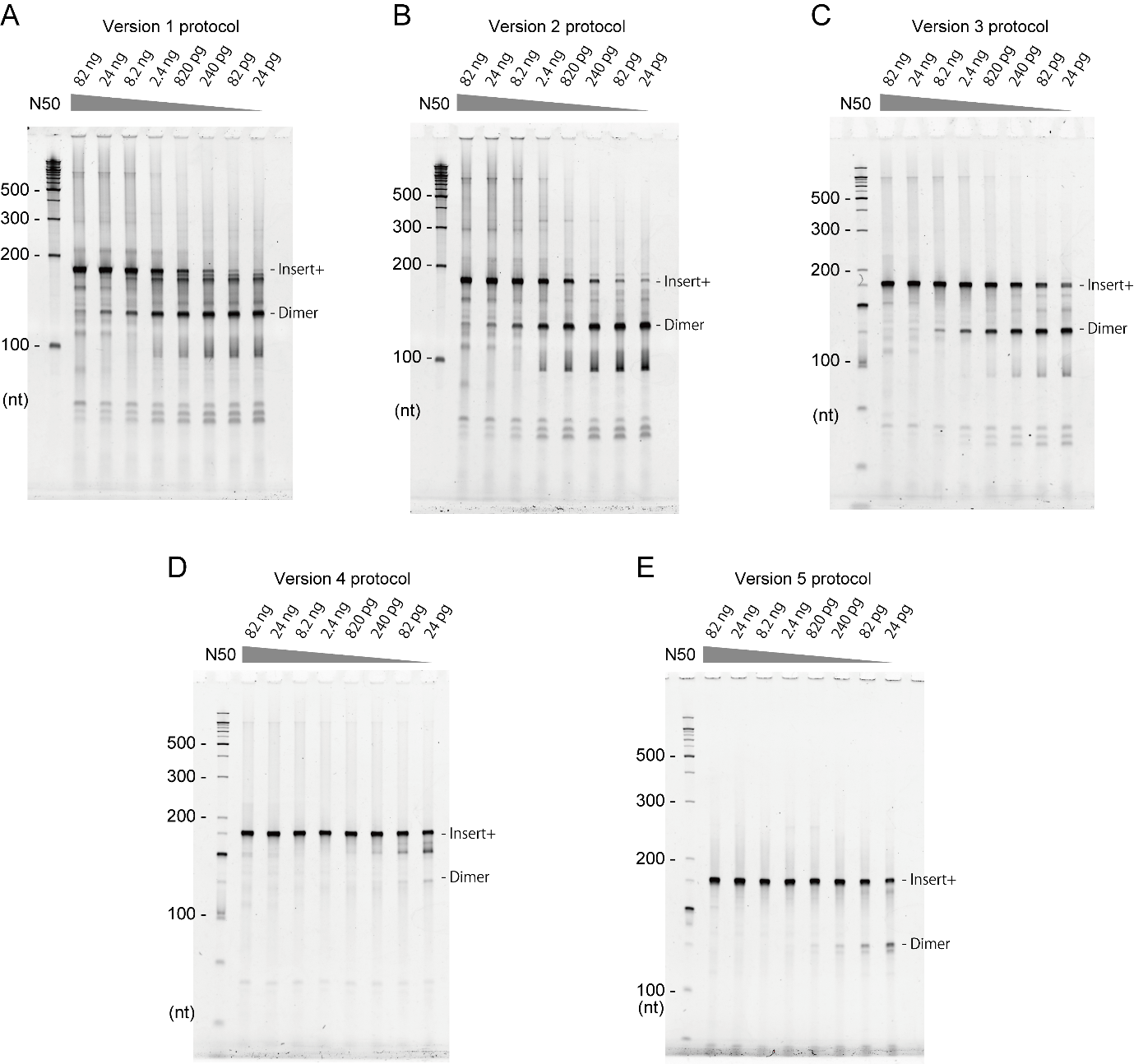


**Supplementary Figure S12**. **Improvement of adaptors for dimer-less library preparations (The original gel images for Supplementary Figure S2).** A. The original gel image for Supplementary Figure S2B. B. The original gel image for Supplementary Figure S2C. C. The original gel image for Supplementary Figure S2D. D. The original gel image for Supplementary Figure S2E. E. The original gel image for Supplementary Figure S2F.

**Supplementary Methods**

**Supercoil relaxation assay (Topoisomerase activity assay)**

A 20-µL reaction containing 180 ng (100 fmol) of pUC19 DNA in 50 mM Tris-HCl, pH 8.0, and 75 mM NaCl was supplemented with either 0, 10, 20, or 40 pmol VTopoI and incubated at room temperature for 10 min. The reaction was then supplemented with 2 µL of 10% (w/v) sodium dodecyl sulfate (SDS) and loaded on 1% (w/v) agarose gel (TAE buffer system). After electrophoresis, the gel was stained with SYBR Gold gel stain (Thermofisher Scientific), and the gel image was captured using the ChemiDock imaging system (Bio-rad Laboratories, Hercules, CA).

**Investigation of ligation specificity of the TOPO-activated adaptor**

A 50-µL reaction containing 25 pmol each of TOPO-substrate F and TOPO-substrate R (Supplementary Table S1) in 0.5× TACS Buffer, 1× ExTaq Buffer, 200 µM ATP, 200 µM of each dNTP, and 20% (w/v) PEG6000 was incubated at 95 °C for 3 min and then 55 °C for 5 min. After cooling the reaction temperature to less than 37 °C, 5 µL of 0.5 M EDTA solution (pH 8.0) and 150 ng VOC were added, after which the reaction was incubated at room temperature for 15 min. The reaction was then supplemented with 45 µL of Buffer B2 and 5 µL of proteinase K and incubated at 50 °C for 15 min. The reaction was supplemented with 100 µL of isopropanol, and the mixture was loaded onto a ZymoSpin column (Zymo Research). The column was washed with Buffer PE (Qiagen), and purified DNA was eluted with 10 µL of 10 mM Tris-HCl, pH 8.5. The purified DNA was loaded on 10% acrylamide gel (TBE buffer system). After electrophoresis, the gel image was captured using the ChemiDock imaging system.

**Sequence analysis of ancient DNA**

Sequenced reads were first processed using fastp with the following options.

For Kapa Hyper Prep Kit library:

--adapter_sequence=AGATCGGAAGAGCACACGTCTGAACTCCAGTCAC

--adapter_sequence_r2=AGATCGGAAGAGCGTCGTGTAGGGAAAGAGTGT

--merge

For TACS-TOPO library

-f 2

-F 4

--adapter_sequence=AAGGGGATCGGAAGAGCACACGTCTGAACTCCAGTCAC

--adapter_sequence_r2=AAAGATCGGAAGAGCGTCGTGTAGGGAAAGAGTGT

--merge

The merged reads were mapped with BWA using the “mem” option against the human reference genome hg19. Finally, mapped alignments in the SAM format were served for the analysis with mapDamage.

**Supplementary Sequences**

**The artificially synthesized gene encoding VTopoI** (The BamHI and EcoRI sites are underlined)

GGATCCATGCGTGCATTGTTCTACAAAGACGGGAAACTTTTTACCGACAATAATTTCCTG

AATCCGGTAAGTGACGACAATCCAGCGTATGAAGTTTTGCAGCACGTGAAAATTCCGACC

CATCTTACCGATGTTGTGGTTTATGAACAAACGTGGGAAGAAGCTCTGACCCGTCTGATT

TTCGTTGGCAGTGATTCAAAAGGTCGAAGACAGTACTTCTACGGGAAAATGCATGTCCAG

AATCGCAACGCAAAACGGGATCGCATATTTGTGCGCGTATATAACGTGATGAAGCGCATC

AATTGTTTCATCAACAAAAACATCAAAAAGAGCTCAACTGATTCGAACTATCAGTTAGCC

GTGTTTATGCTCATGGAGACCATGTTCTTCATTCGCTTTGGCAAAATGAAGTACCTCAAA

GAGAATGAAACGGTAGGCTTACTGACCTTAAAGAACAAACACATTGAAATCAGCCCTGAT

GAGATTGTGATCAAATTTGTGGGTAAAGACAAAGTCAGCCATGAATTTGTCGTCCACAAA

TCCAATCGCCTGTATAAACCGCTGCTGAAACTCACTGACGATTCTTCCCCAGAAGAATTT

CTGTTTAACAAACTGTCTGAACGTAAGGTGTACGAGTGCATTAAGCAATTCGGTATTCGT

ATTAAGGATCTGCGTACGTATGGTGTCAACTATACGTTTCTGTACAACTTTTGGACCAAC

GTAAAGAGCATATCTCCCTTACCTAGTCCGAAAAAACTGATTGCGCTTACCATCAAACAG

ACAGCGGAAGTTGTTGGACATACACCGAGCATCTCAAAACGTGCCTATATGGCTACGACT

ATCCTAGAAATGGTGAAAGACAAAAATTTCCTGGATGTTGTTTCGAAAACGACCTTTGAT

GAGTTTTTGTCGATTGTCGTGGATCATGTGAAAAGCTCCACAGATGGCGAATTC

**The PCR-amplified gene encoding T4 Polynucleotide kinase cloned into pColdI** (The BamHI and EcoRI sites are underlined, and primer sequences used to amplify the gene are italicized)

*ATGGGTCGCGGATCCATGAAAAAGATTATTTTGAC*TATTGGCTGTCCTGGTTCTGGTAAG

AGTACTTGGGCTCGTGAATTTATTGCTAAGAATCCCGGGTTTTATAATATCAATCGTGAT

GACTATCGCCAATCTATTATGGCGCATGAAGAACGCGATGAGTACAAGTATACCAAAAAG

AAAGAAGGTATCGTAACTGGTATGCAGTTTGATACAGCTAAAAGTATTCTGTACGGTGGC

GATTCTGTTAAGGGAGTAATCATTTCAGATACTAACCTGAATCCTGAACGTCGCCTAGCA

TGGGAAACTTTTGCCAAAGAATACGGCTGGAAAGTTGAACATAAAGTGTTTGATGTTCCT

TGGACTGAATTGGTTAAACGTAACTCAAAACGCGGAACTAAAGCAGTACCAATTGATGTT

TTACGTTCAATGTATAAAAGCATGCGAGAGTATCTCGGTCTTCCAGTATATAATGGGACT

CCTGGTAAACCAAAAGCAGTTATTTTTGATGTTGATGGTACACTAGCTAAAATGAATGGT

CGTGGTCCTTATGACCTTGAAAAATGCGATACCGATGTTATCAATCCTATGGTTGTTGAA

CTGTCTAAGATGTATGCTCTTATGGGTTATCAAATCGTAGTCGTTTCAGGTCGTGAAAGT

GGAACTAAAGAAGACCCAACGAAATATTATCGTATGACCCGTAAATGGGTTGAGGACATT

GCTGGCGTTCCATTAGTTATGCAATGTCAGCGCGAACAAGGCGATACCCGTAAAGACGAT

GTAGTTAAAGAAGAAATTTTCTGGAAACACATTGCACCGCATTTTGACGTGAAATTAGCT

ATTGATGACCGAACTCAAGTAGTTGAAATGTGGCGTCGTATCGGTGTTGAATGCTGGC*AA*

*GTCGCTTCGGGAGATTTTGAATTCGAGCTCCGT*
